# DNA analysis of elasmobranch products originating from Bangladesh reveals unregulated elasmobranch fishery and trade on species of global conservation concern

**DOI:** 10.1371/journal.pone.0222273

**Published:** 2019-09-25

**Authors:** Alifa Bintha Haque, Sudipta Arka Das, Aparna Riti Biswas

**Affiliations:** 1 Department of Zoology, University of Dhaka, Dhaka, Bangladesh; 2 Department of Zoology, University of Oxford, Oxford, United Kingdom; 3 Department of Biochemistry and Molecular Biology, University of Dhaka, Dhaka, Bangladesh; National Zoological Park, UNITED STATES

## Abstract

Trade involving elasmobranch products in Bangladesh is a four-decade-long practice in large scale and there is little understanding of its impact on species composition, population, and subsequent conservation. Capacity for monitoring and identification is lacking in landing and shark processing centres. A rapid survey and collection of tissue samples were performed in three landings and nine shark processing centres between 2016 and 2017 in the south-eastern coastal region of Bangladesh. Sequencing for a 707-bp fragment of the mitochondrial cytochrome c oxidase subunit I (COI) gene was used to assess the taxonomic status and species composition from 71 elasmobranch tissue samples collected from the shark processing centre only. Good quality COI sequences were obtained for 34 specimens representing 21 species—the majority of which are threatened with extinction. A total of ten species of sharks (*Carcharhinus brevipinna*, *C*. *amboinensis*, *C*. *leucas*, *C*. *sorrah*, *C*. *amblyrhynchoides*, *Chiloscyllium burmensis*, *Galeocerdo cuvier*, *Rhincodon typus*, *Scoliodon laticaudus*, and *Sphyrna lewini*), eleven species of rays (*Aetomylaeus maculatus*, *Gymnura poecilura*, *Mobula mobular*, *M*. *kuhlii*, *Neotrygon indica*, *Pateobatis uarnacoides*, *Rhinoptera javanica*, and *R*. *jayakari*), including three species of guitarfish (*Glaucostegus granulatus*, *G*. *obtusus*, and *G*. *typus*), were identified. Four species (14.7% of samples) were found to be listed in the Convention on International Trade in Endangered Species of Wild Fauna and Flora (CITES) in Appendix II. Sixteen species (59% of the specimens) were threatened with extinction according to IUCN Red List, whereas 41% were data deficient or not assessed. The results have important implications for the management of regional fisheries and the conservation of elasmobranchs as they 1) represent a preliminary understanding of elasmobranch diversity in trade; 2) depict a lack of awareness and monitoring; and 3) demonstrate a need for urgent monitoring and regulation of elasmobranch trade in Bangladesh.

## Introduction

Estimates suggest that a quarter of shark and ray species are now threatened with extinction primarily driven by unsustainable fisheries, unprecedented bycatch, habitat destruction and illegal unreported and unregulated (IUU) practices globally [1–15]. Despite their threatened statuses, elasmobranchs still represent a large component of global fisheries [11–14]. Fisheries target elasmobranchs for their meat and fins [1–10]. Many Asian countries, and especially China, have become a hub for elasmobranch trade [3], in part due to a lack of national legislation that would protect threatened shark and ray species. China, along with an array of other Asian countries (Sri Lanka, India, Bangladesh, Indonesia, Malaysia, Maldives, Myanmar, Sri Lanka, and Thailand) are either importing, exporting, or both [13–19], thus making Asia and adjacent areas the trade hub for elasmobranchs. Unfortunately, these fisheries are largely unregulated and data on species composition is extremely limited [20]. Conservation measures to protect elasmobranchs from overexploitation are gaining global priority [9] with a focus on trade regulation [17]. This is hindered by the inability of authorities to track species-specific catch and trade in data-poor regions. Trade monitoring becomes more complicated when the origin of the products is untraceable after shark products are harvested, cut, and processed in unmonitored market chains [17, 18]. Hence, DNA barcoding has been used as an efficient method to monitor traded products in several studies addressing this issue [3].

Little is known about the elasmobranch communities of the Bay of Bengal [21] due to a lack of species-specific research regarding their ecology, biology, habitats, trade, and species composition [19]. India has reported 117 species of sharks from 23 families, 119 species of rays from 18 families including 11 species of guitarfish and 5 species of sawfish in Indian waters. Of these species identified, 41 still require confirmation and 27 are considered questionable by the authors [22]; Bangladesh has reported only 30 species of sharks from 8 families; and 29 species of rays from 7 families, including 5 species of guitarfish and 3 species of sawfish [18, 21] with confirmed records of additional species still unpublished [Haque in prep.].

Bangladesh is not on the global map as a shark product producer even though substantial catch, trade, and IUU have been reported [18–27]. The national law of Wildlife (protection and security) Act, 2012 in Bangladesh prohibits killing 29 species of elasmobranchs under two schedules. Species under schedule I and II are designated as ‘protected animals’ where the harming of these is designated as a crime punishable with fines and imprisonment. Moreover, Bangladesh is a signatory of the Convention on International Trade in Endangered Species of Wild Fauna and Flora (CITES). Any primarily commercial trade of species listed under CITES Appendix I is prohibited and requires a permit. Under CITES Appendix II regulations, export and trade of the species listed requires a permit ensuring that the trade is sustainable and not detrimental. However, several thousand kilograms of shark and guitarfish products have been reported to be exported through a port not regulated by national customs authority from Bangladesh [18]. Between 2012 and 2015, elasmobranch exports worth an estimated USD 250,494 have been reported from just one processing center out of several present in the south-eastern region [18] indicating lack of monitoring.

Reporting on elasmobranch catch in the landing sites is inefficient and resources are lacking in Bangladesh. The current method of accounting for all elasmobranch landings in one box entitled ‘sharks, skates, and rays’ and fin trade accounting with fish maw, reveals that the national fisheries statistics are under-reporting the extent of catch, trade, species composition, and diversity. The clear unsustainability of elasmobranch trade in the region justifies taking immediate action to mitigate these threats.

Comprehensive surveys of elasmobranch diversity and trade have been conducted in areas of intense fishing pressure including the Philippines, Indonesia, Taiwan, and the Middle East [10, 13, 15, 28, 29]. Bangladesh represents a conspicuous gap in global surveys in regard to species diversity in catch and trade. The current study was a preliminary effort to identify the species composition of shark and ray products originating from Bangladesh for domestic and international markets using molecular techniques. It reports important metrics regarding the existing trade of globally and nationally protected species in Bangladesh, which fills data gaps, and also recommends studies in this region to promote evidence-based management.

## Methods

**Fig 1:**
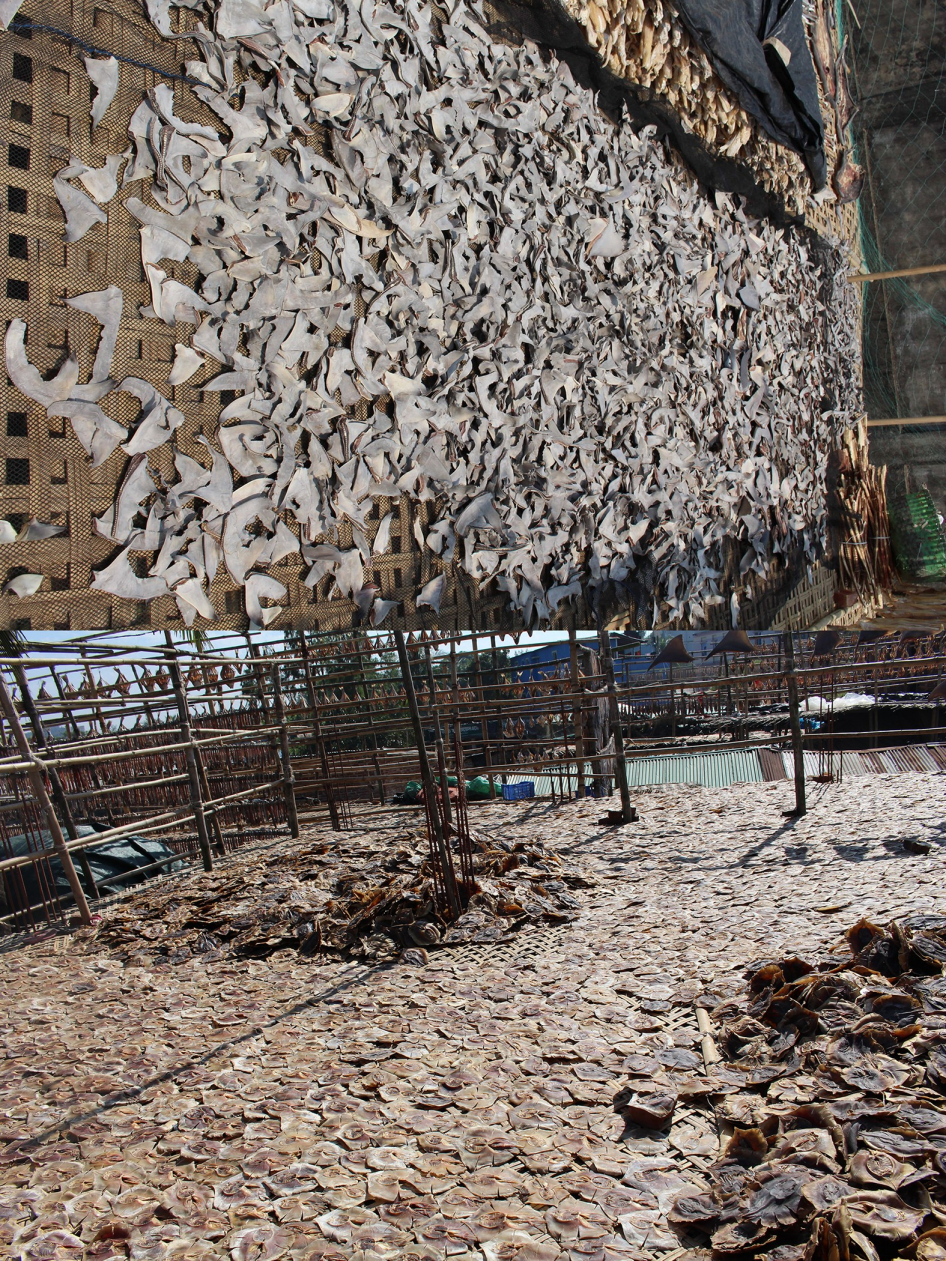
a & b. Shark fins and dried small rays in a processing centre in Cox’s Bazar on March, 2017; drying to be distributed to the national and international markets. It is a common summer day in the processing centres.

**Fig 2:**
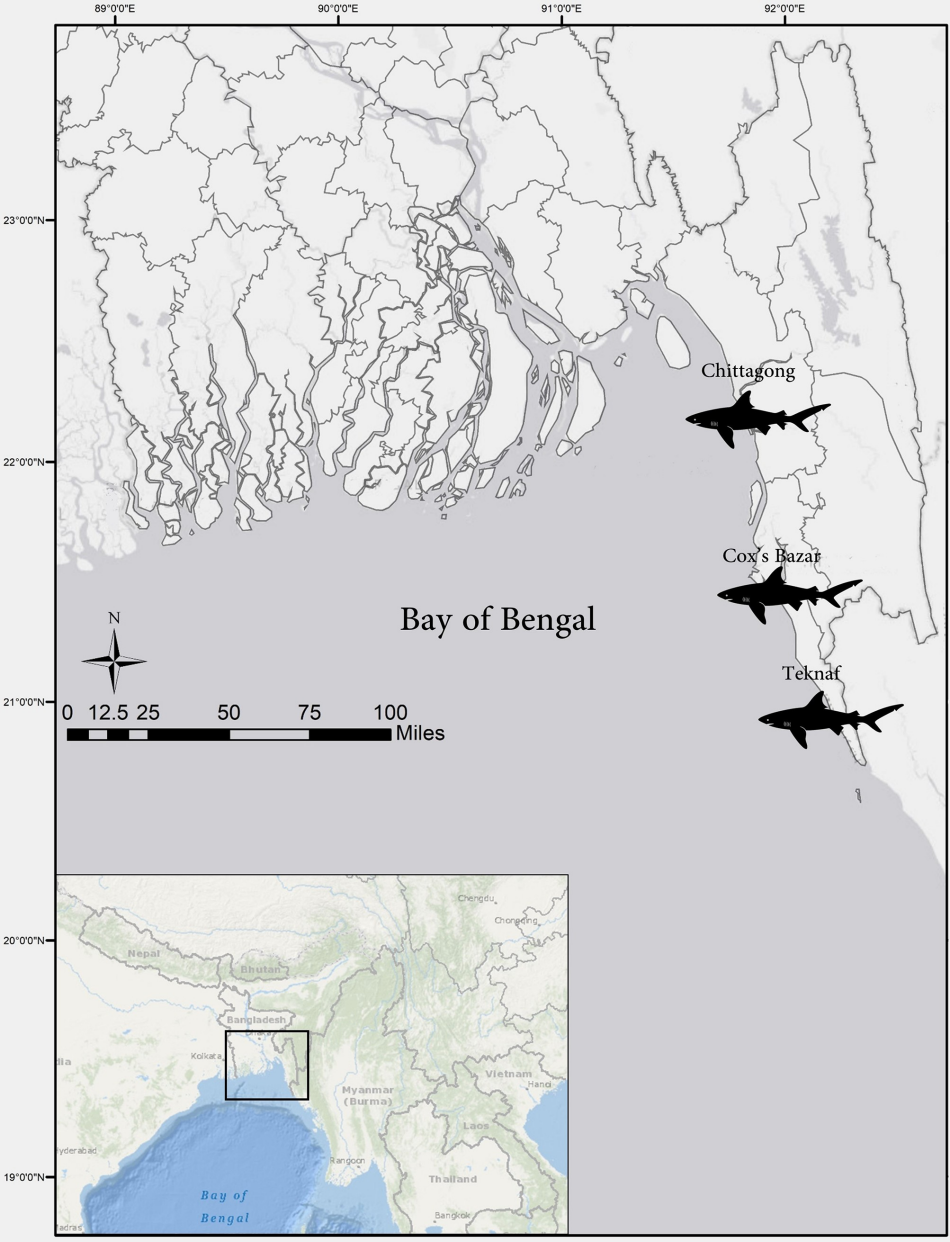
Study area (inset) and study sites in the southwestern coastal region of Bangladesh. Sharks: Chittagong, Cox’s Bazar, Teknaf: where the elasmobranch tissue samples were collected from different processing centres between June 2016 and June 2017.

A total of 556 samples of harvested fresh, dried, semi-dried and salted meat, fins, skin, and mobulid gill plates of sharks and rays were collected and preserved in 98% ethanol and then kept in -20° C until further analysis. Samples were collected from products ready to be traded to Myanmar, through Teknaf, between June 2016 and June 2017. Approximately five to ten samples were collected (one to two samples from each pile of products on each field visit, 18 visits) from each of the four exclusive shark processing centres in Cox’s Bazar (21.452075, 91.968270) (Fig 1A & 1B). However, samples were also opportunistically collected from five centres in Chittagong (22.319428, 91.838783) and Teknaf (20.857906, 92.297397) (Fig 2). This was done carefully to avoid sample redundancy by collecting fewer samples (n = 1 to 2) from one pile of products. Help was taken from processing centre workers to avoid obtaining multiple samples from the same species when possible. Moreover, when samples were collected from fins; caudal fin clips were preferentially collected to avoid collecting from the same species. For analysis, a subset of 71 samples (three to four samples from each visit) was randomly selected due to limited resources. These analyzed samples were otherwise impossible to identify morphologically after processing.

Permission to collect DNA samples was taken from the Bangladesh Forest Department and the Ministry of Environment, Forest and Climate Change. The Bangladesh Forest Department is the authoritative body to grant such research on nationally protected and CITES-listed species.

Cubes of tissue of approximately 2mm in size were subsampled from the meat, skin, or fin clippings preserved in ethanol for DNA extractions. The total genomic DNA extraction was done using ReliaPrep™ gDNA Tissue Miniprep System (Promega, USA) following the manufacturer’s instructions. The DNA quality was assessed following electrophoresis through 1% agarose stained with ethidium bromide and visualized under UV light.

The cytochrome c oxidase subunit I gene (COI) was amplified using a combination of the primers FishF1 and FishR1 [30]. Amplification PCR was done using GoTaq® G2 Master Mix (Promega). PCR conditions were 95°C for 2 min, 35 cycles of (94°C for 30 s, 60°C for 30 s, 72°C for 1 min), 72°C for 10 min, 4°C hold [31]. Afterward, PCR agarose gel electrophoresis was performed to confirm successful amplification. Amplified product was purified using Wizard^®^ PCR Preps DNA Purification System (Promega).

Purified PCR product was sequenced using BigDye Terminator v3.1 Cycle Sequencing Kit (Thermo Fisher Scientific) and ABI 310 Genetic Analyzer (Applied Biosystems). Sequence data were assembled and FASTA data files were created using SeqScape Software (Applied Biosystems). FASTA sequences were compared with the National Center for Biotechnology Information (NCBI) Database [https://www.ncbi.nlm.nih.gov/] and closely matched sequences from the search were downloaded. Species-level identification was performed using the Basic Local Alignment Search Tool (BLAST) algorithm of the NCBI [32]. A top species match (megablast, nr/nt, taxid:7778) was identified with a sequence similarity of at least 99%. A Neighbor-joining tree with closely matched sequences for each species was constructed using BLAST tree view (S1 Fig).

For more robust results, second species identification was conducted using BoLD database (http://www.boldsystems.org/). Sequences closely matched from the BLAST search were downloaded from the BoLD database. Sequences mined from NCBI were avoided when possible. Sequence alignment and Phylogenic tree were constructed from the downloaded sequences using ‘Phylogeny.fr’ toolset [33–38]. Phylogeny.fr is the platform that transparently chains programs relevant to phylogenetic analysis in a pipeline. Three steps of the pipeline–alignment, alignment refinement, and phylogeny were used. Phylogeny.fr allows a choice of several algorithms in each step (http://www.phylogeny.fr/documentation.cgi). MUSCLE 3.8.31, Gblocks 0.91b and PhyML 3.1/3.0 aLRT algorithms were used for alignment, alignment refinement, and phylogeny respectively. Pairwise distance table was generated using MEGA-X from aligned sequences (S1 Table). The phylogenic tree was visualized using ‘iTOL’ [39].

## Results

COI barcode sequences were obtained for 34 samples with sequence lengths varying between 281 and 650 bp (average length 577 bp) (Table 1). The remaining samples either failed to produce PCR bands or produced nonspecific sequences.

**Table 1:** Total number (n) and percentage of total (%) species recorded within the study from Bangladesh with their IUCN Red List status and their assessment dates including CITES listing and status in Wildlife (protection and security) Act, 2012, Bangladesh. (EN Endangered; NT Near Threatened; VU Vulnerable; DD Data Deficient; LC Least Concern).

Sl. No.	Family	Species	Common Name	No. of samples	% in sample	CITES listing	IUCN status	National protection^1^
**Sharks**								
	Family	Species	Common name	No. of Samples	% in samples	CITES listing	IUCN status	National protection*
1	Sphyrnidae	*Sphyrna lewini*	Scalloped Hammerhead	2	5.88	APP.II	EN (2007)	Schedule I
2	Rhincodontidae	*Rhincodon typus*	Whale Shark	1	2.94	APP.II	EN (2016)	Schedule I
3	Carcharhinidae	*Carcharhinus brevipinna*	Spinner Shark	1	2.94		NT (2005)	NP**
4		*Carcharhinus leucas*	Bull Shark	1	2.94		NT (2005)	NP
5		*Carcharhinus sorrah*	Spottail Shark	3	8.82		NT (2007)	Schedule I
6		*Carcharhinus amblyrhynchoides*	Graceful Shark	1	2.94		NT (2005)	NP
7		*Galeocerdo cuvier*	Tiger Shark	1	2.94		NT (2005)	Schedule I
8		*Scoliodon laticaudus*	Spadenose Shark	1	2.94		NT (2005)	Schedule I
9		*Carcharhinus amboinensis*	Pigeye Shark	7	20.59		DD (2005)	NP
**10**	Hemiscylliidae	*Chiloscyllium burmensis*	Burmese Bamboo Shark	2	5.88		DD (2008)	NP
**Rays**								
11	Myliobatidae	*Mobula mobular*	Giant Devil Ray	1	2.94	APP.II	EN (2014)	NP
12		*Mobua kuhlii*	Shortfin Devil Ray	1	2.94	APP.II	DD (2007	NP
13		*Aetomylaeus maculatus*	Mottled Eagle Ray	1	2.94		EN (2006)	NP
14		*Rhinoptera javanica*	Javanese Cownose Ray	1	2.94		VU (2006)	NP
15		*Rhinoptera jayakari*	Oman Cownose Ray	2	5.88		-	NP
16	Gymnuridae	*Gymnura poecilura*	Longtail Butterfly Ray	2	5.88		NT (2006)	Schedule II
17	Dasyatidae	*Neotrygon indica*	Blue-spotted Maskray	2	5.88		-	NP
18		*Pateobatis uarnacoides*	Bleeker's Whipray	1	2.94		VU (2004)	NP
**Guitarfish**								
19	Glaucostegidae	*Glaucostegus granulatus*	Sharpnose Guitarfish	1	2.94		VU (2006)	Schedule I
20		*Glaucostegus obtusus*	Widenose Guitarfish	1	2.94		VU (2006)	NP
21		*Glaucostegus typus*	Giant Shovelnose Ray	1	2.94		VU (2003)	NP

*Wildlife (protection and security) Act, 2012, Bangladesh

**NP = Not protected

After catching the sharks, harvested products are poorly preserved and an array of processing techniques are used such as air-dried in the open and either salted or unsalted. Products are then mixed with other species which confounds the identification process. These preservation methods paired with the use of poor packaging methods until the point of sale, allows for deterioration in tissues and degradation in DNA which, in turn, can affect the sequencing success. Failed PCR might have been due to DNA degradation.

Nonspecific sequences were mostly microbes and not included in the sequences submitted. Within the 34 samples identified using NCBI’s BLAST tool, there was a sequence similarity value greater than 99% in all cases.

**Fig 3:**
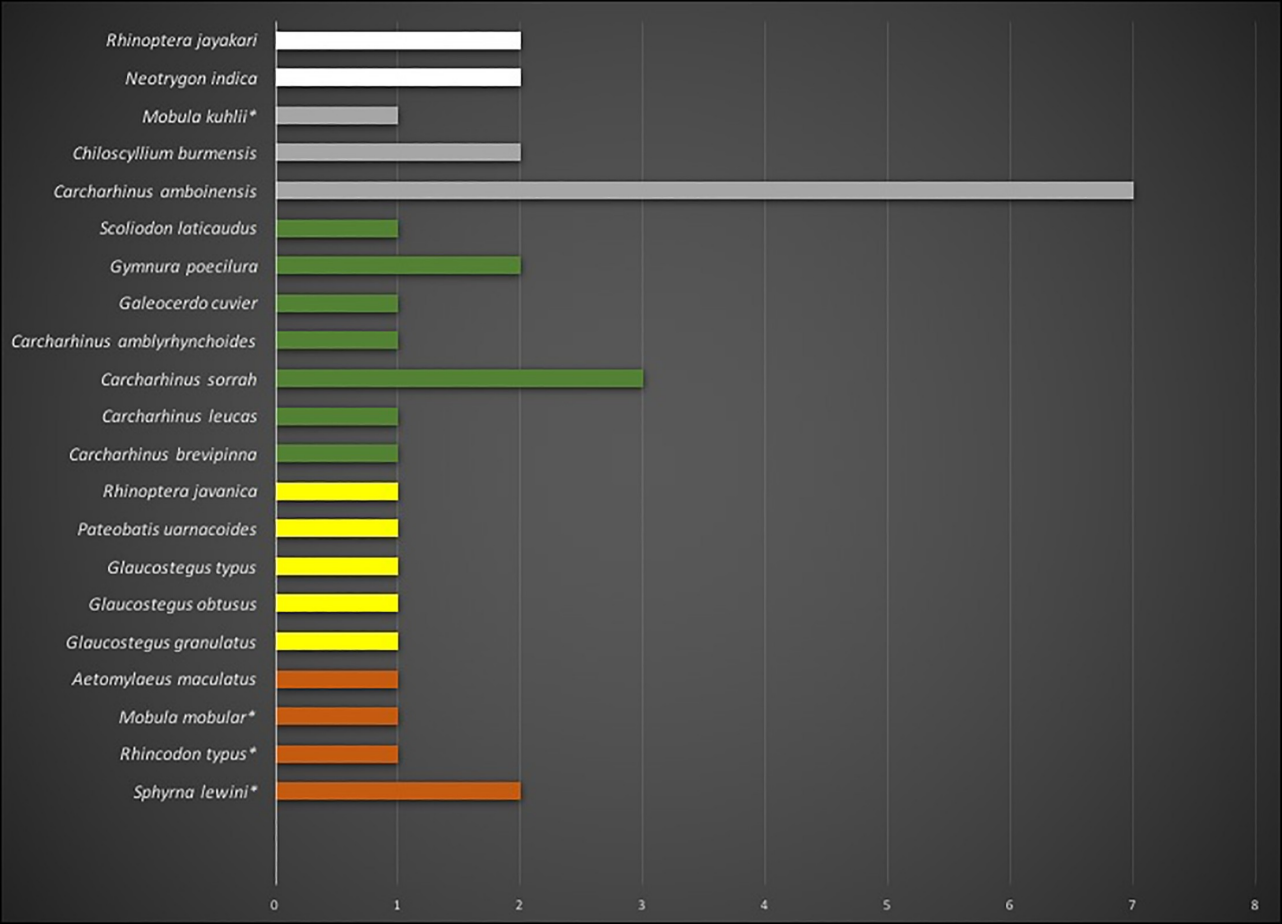
Species identifications for 34 elasmobranch products (dried, semi-dried, or salted meat and fins). Bar length represents abundance; color and order (Y-axis) correspond to IUCN Red List of Threatened Species status (Orange: EN, yellow: VU, green: NT, gray: DD and white: not assessed). Species currently listed in CITES Appendix II in 2017 are marked with asterisks.

Within the current study, a total of 21 species of elasmobranchs were identified from the traded products. The species richness is extremely high and a number of specimens of each species ranged from one to seven which is particularly low considering the low number of samples which were analyzed. A total of ten species of sharks and eleven species of rays, including two species of devil rays and three species of guitarfish, were identified (Table 1). More than half of the species are threatened with extinction according to the IUCN Red List of Threatened Species. Among the species identified, 14.7% are Endangered, 14.7% are Vulnerable, 29.41% are Near Threatened, 29.41% are Data Deficient, and 11.76% are not assessed by the IUCN Red list threatened category. Four species (14.7%) are listed in Appendix II by CITES (e.g., *Sphyrna lewini*, *Rhincodon typus*, *Mobula mobular*, & *M*. *kuhlii*) (Fig 3).

Amongst all these sampled species, seven species are protected by the Wildlife (protection and security) Act, 2012 of Bangladesh (Table 1) which makes the collection of those products a punishable crime. The richness of species is very high given the small number of samples analyzed. *Carcharhinus amboinensis* was the most common species found in the collected samples (20.59%), whereas *C*. *sorrah* is the second highest (8.82% each), followed by *S*. *lewini* and *C*. *burmensis* (5.88% each).

However, there were two discrepancies between databases for identifications using both NCBI’s BLAST tool (S1 Fig) and the BoLD database (S2 Fig; S2 Table). These were *Glaucostegus typus* (MH841985) and *Mobula mobular* (MH842002). In the BoLD database tree, results show that the species are closer to *G*. *obtusus* and *M*. *japonica*, respectively. As the authors worked with samples obtained from processing centers, morphologic identification wasn’t possible to generate conclusive comments. Moreover, there are very few publicly available sequences for *M*. *mobular* to validate the results, revealing the existing limitations of species identification purely based on barcode. Sequencing whole mitochondria can provide a better resolution towards such problems.

This study recorded *Chiloscyllium burmensis and Rhinoptera jayakari* for the first time in Bangladesh. This is presumably due to inability of identification to species level in previous studies.

## Discussion

Trade on biological resources proves to be a threat for many species globally, leading to unsustainable capture rates from the wild. The growing demand for aquatic animals, especially fish, has driven many targeted and non-targeted fishing practices to either extirpate populations or cause great declines in population levels. Trade on elasmobranch species is a long-standing practice in the coastal areas of Bangladesh [18, 26, 21]. Population declines have already been caused, likely due to this practice being unregulated and unmonitored.

Although the current study deals with a small number of samples, it still provides much-needed information for a data-poor region of high conservation concern, the Bay of Bengal. While the twenty-one identified species are not a complete representation of the traded species composition, this study shows that several nationally and internationally protected species with extinction risk are present in traded products.

The challenges to deal with the diverse trade dynamics at local, regional, and global scales are identified to be: 1) Lack of national protection through legislation and enforcement; 2) lack of conformity amongst the national, regional and global protection levels; 3) lack of efficient monitoring techniques and monitoring capacity; and 4) lack of awareness among the fishers and traders [Haque in prep.].

### Need for better legislation and enforcement

Bangladesh is a CITES signatory, and therefore has agreed to ban the unpermitted trade of CITES-listed species internationally. Twelve species of shark and all *Mobula* spp. have been listed under CITES Appendix II and all *Pristis* spp. are listed under CITES Appendix I. Moreover, 29 species are prohibited to be caught and traded on by the national law: Wildlife (Conservation and Security) Act, 2012 [18]. Failure to abide by the law is punishable by financial penalties and imprisonment. However, these have not been a sufficient incentive to regulate trade alone where monitoring and enforcement are lacking [Haque unpubl. data].

This study identified trade exists for eight species (Table 1) of sharks. It reports trade on two globally important shark species *Rhincodon typus* and *S*. *lewini* listed under CITES Appendix II. Furthermore, both Data Deficient *Carcharhinus amboinensis* and Neat Threatened *C*. *brevipinna* and *C*. *leucas* were also reported to be commonly landed, however, none of them are protected either by national law or CITES. *Chiloscyllium burmensis* was also reported, which is trashed in Bangladesh owing to no domestic or international value, hence the most neglected shark species (e.g., bamboo sharks). The study also revealed a dearth of species-specific studies especially on devil rays, sawfish, and guitarfishes in Bangladesh although several studies now reported their trade from here [18, 40]. Eleven species (Table 1) of rays from the sampled products have been identified. However, these products were prepared either for domestic or international trade. Whereas the majority of the shark products and some of the devil rays’ parts (e.g., gill rakers) were confirmed to be traded internationally, the rest of the samples’ destination could not be evaluated.

Amongst the sampled ray products, only *M*. *mobular* and *M*. *kuhlii* have been identified in trade, but other *Mobula* spp. known to range in Bangladeshi waters are likely traded, yet are not represented in this study due to the small number of samples analyzed. *Mobula japanica* has also been reported to be traded in previous studies [18]. Although the most popular product to be traded from devil rays is gill rakers [41], the current study reveals a national market along with international demand for its meat as well. Broadly, trade on protected species has been recorded without any enforcement of the law throughout the market chain.

Many of these species range across country boundaries, and as such, laws protecting them in neighboring courtiers are also of importance. These varying levels of protection and threatened statuses show discrepancies, especially amongst the IUCN Red List, CITES, and the national law of Bangladesh and neighboring countries. Uniformity in legal status amongst neighboring countries would allow for more effective enforcement of wildlife trade laws across political boundaries as part of a concerted approach to save all threatened elasmobranch species.

### Need for better, easier and low-cost monitoring

Effective monitoring improvements are necessary. Bangladesh is a resource-poor country and protection for elasmobranchs by legislation is relatively new (since 2012). Capacity to monitor coast-wide trade is difficult due to the lack of trained officers. Along with that, the lack of easy and rapid mechanisms to identify products and species aggravates the issue. Although a signatory of CITES, implementation of these regulations is confounded by the inability to accurately and efficiently confirm species identifications of exported products in the field and ports. Identification is either impossible or very difficult in processed body parts without proper diagnostic characters. Confirming species identifications can be further complicated as an array of names can be used to designate marketed products which do not correspond with the taxonomic names and apply to multiple morphologically similar species [15, 42]. Laws are being broken due to lack of monitoring and identification capacity of law enforcement bodies, along with a complete lack of awareness amongst the fishers and traders [Haque in prep].

To more accurately identify products, many visual identification guides [43–45] and molecular techniques [46–50] have been developed over the past few years. Several studies have successfully proposed molecular techniques to facilitate the identification of fresh or processed products for market surveillance [3, 15, 17, 28, 51–53]. Hence, alternative cost-effective methods are important to innovate within developing country contexts.

The current study supports the use of molecular techniques to identify an array of protected species, considering the absence of accurate fishery data on elasmobranchs in Bangladesh [54]. Since, there are ample reasons for product misidentification in estimating the most threatened species in traded body parts, this method could be used as an effective tool to better monitor and manage trade throughout the market chain. However, implementing this method requires greater commitment and resources from the authoritative bodies which is a great challenge. This study also acknowledges the resource constraints to sustainably conduct these molecular techniques over a long period and recommends developing effective and low-cost alternatives.

### Recommendations for urgent conservation actions

Being a resource-poor, developing country, alternative methods (e.g. PCR mini barcoding, with greater success in amplifying smaller fragments and degraded DNA cost-effectively [55]) are recommended to be utilized for effective monitoring. Alternative, lower-cost enforcement actions such as capacity and awareness building are especially recommended for more effective monitoring at the landing sites for protected species by local authoritative offices of Bangladesh Forest Department in collaboration with Bangladesh Fisheries Development Corporation. It is recommended to enforce not only the CITES mandates which regulate the international trade of rare and protected species, but also the Wildlife (Conservation and Security) Act, 2012 in Bangladesh to protect against threats posed by national trade.

Amendments to the Wildlife (Conservation and Security) Act, 2012 to incorporate species that are threatened with extinction, yet landed regularly and in abundance, are desperately needed. However, only effective monitoring improvements would not be enough to achieve overall trade regulation. Addressing larger issues such as attaining political support and adequate law enforcement mechanisms, in parallel with approaches that reduce bycatch are strongly recommended.

This study also emphasizes the urgent need for conformity amongst the varying degree of protection and threat status locally, regionally, and globally and recommends localized threat status evaluation for more effective conservation.

Further in-depth research on overall trade especially domestic and international ray trade (Myliobatidae, Dasyatidae, and Glaucostegidae) and their impact on overall elasmobranch conservation needs special attention as this has been shadowed by the global interest focusing on shark fin trade. It is also necessary to achieve a holistic understanding of local, regional, and global catch and trade data, route and precise destination with species composition.

Finally, coast-wide campaigns for behavioral change in the reduction of domestic demands and international trade and implementation of by-catch mitigation strategies need to be given utmost importance. However, discussing these management actions is beyond the capacity of this paper.

## Conclusion

The results of the study have important ramifications for elasmobranch species status evaluation and management for a range of geographical scales. Although threatened classifications are based on global assessments, the regional statuses of species can vary geographically, and therefore the threats might be much higher than perceived for localized populations. Bangladesh is an important site for many globally important species and, as such, it is recommended to urgently conduct more comprehensive studies using a mixed-method approach of rigorous surveys in the landing sites to decipher catch trends and trade with an emphasis on CITES-listed, Data Deficient, and species threatened with extinction.

## Supporting information

S1 Fig Molecular species identification using the mitochondrial COI gene for individual samples (n = 34). Neighbour-joining tree of COI gene of supplied specimens and related sequences downloaded from NCBI GenBank are shown individually for each sample. Bootstrap values are shown on each branch.
(PDF)

S2 Fig Maximum likelihood tree based on COI sequences from related samples in ‘boldsystems.org‘ public data portal. *Glaucostegus typus* (accession number: MH841985) and *Mobula mobular* (accession number: MH842002) shows discrepancies with NCBI BLAST based molecular species identification (See supporting information S2 Fig).
(PDF)

S1 Table Pairwise distance table. (XLS)

S2 Table Comparative results on identification of specimens using two different databases with their accession numbers. (DOCX)
