# Supplementary figures and images for: DNA analysis of elasmobranch products originating from Bangladesh reveals unregulated elasmobranch fishery and trade on species of global conservation concern
Source: PLoS One. 2019 Sep 25;14(9):e0222273. doi: 10.1371/journal.pone.0222273 (PMC6760772; doi:10.1371/journal.pone.0222273)

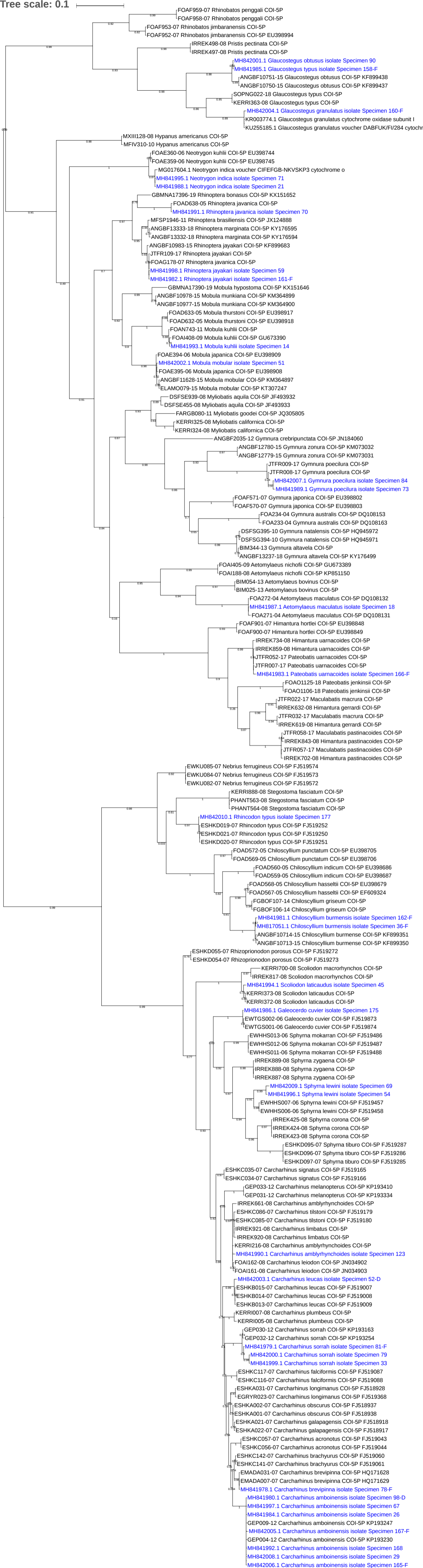

Supplement: S1 Fig — Neighbour-joining tree of COI gene of supplied specimens and related sequences downloaded from NCBI GenBank are shown individually for each sample. Bootstrap values are shown on each branch. (PDF) [file pone.0222273.s001.pdf]

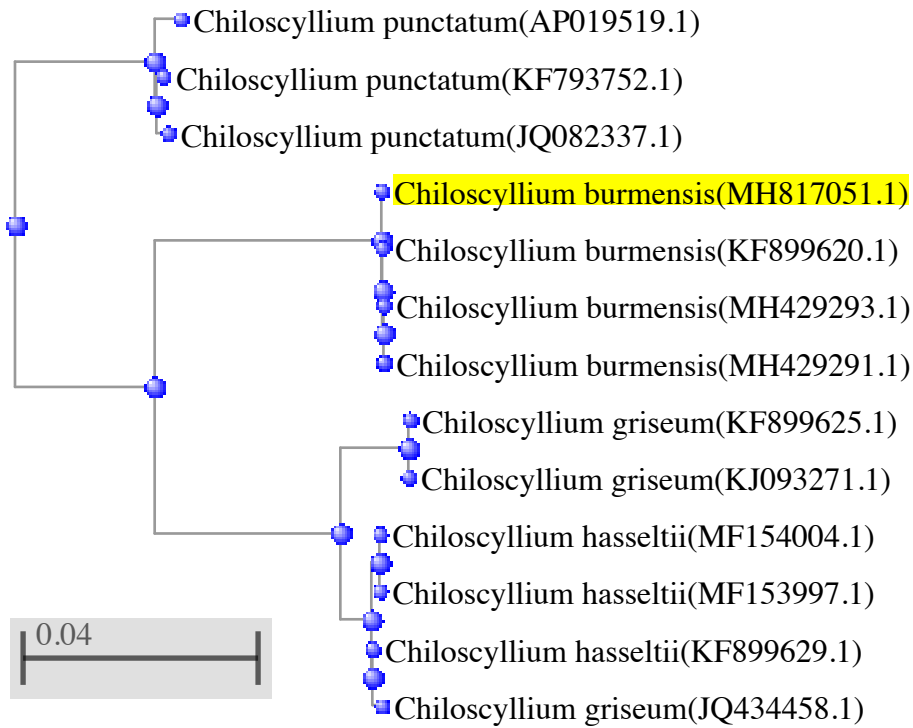

1.

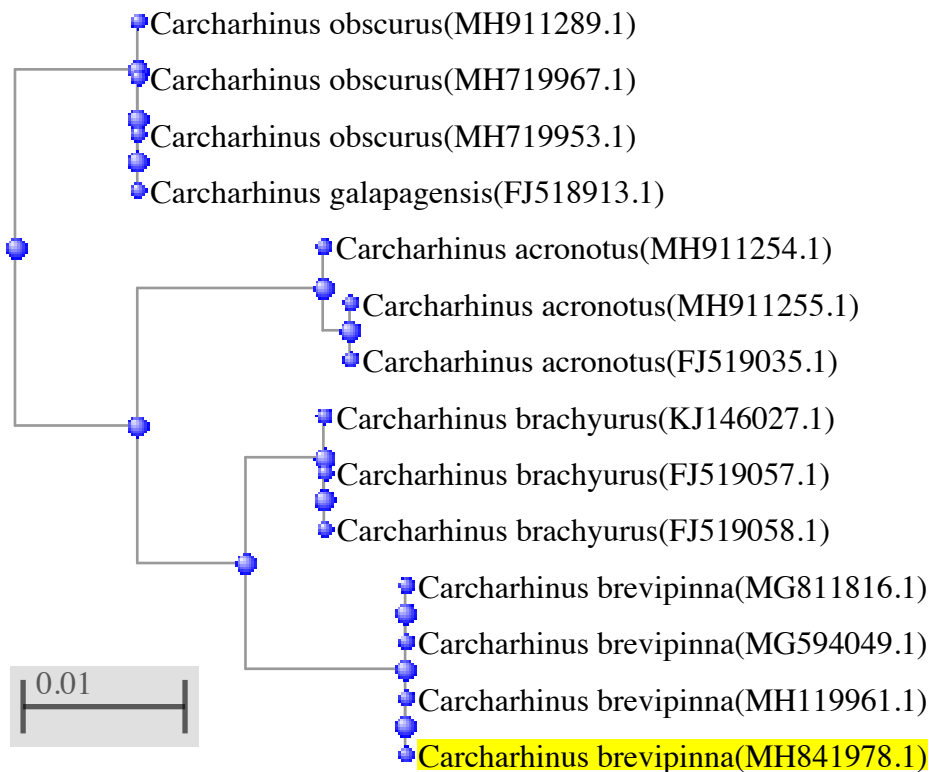

2.

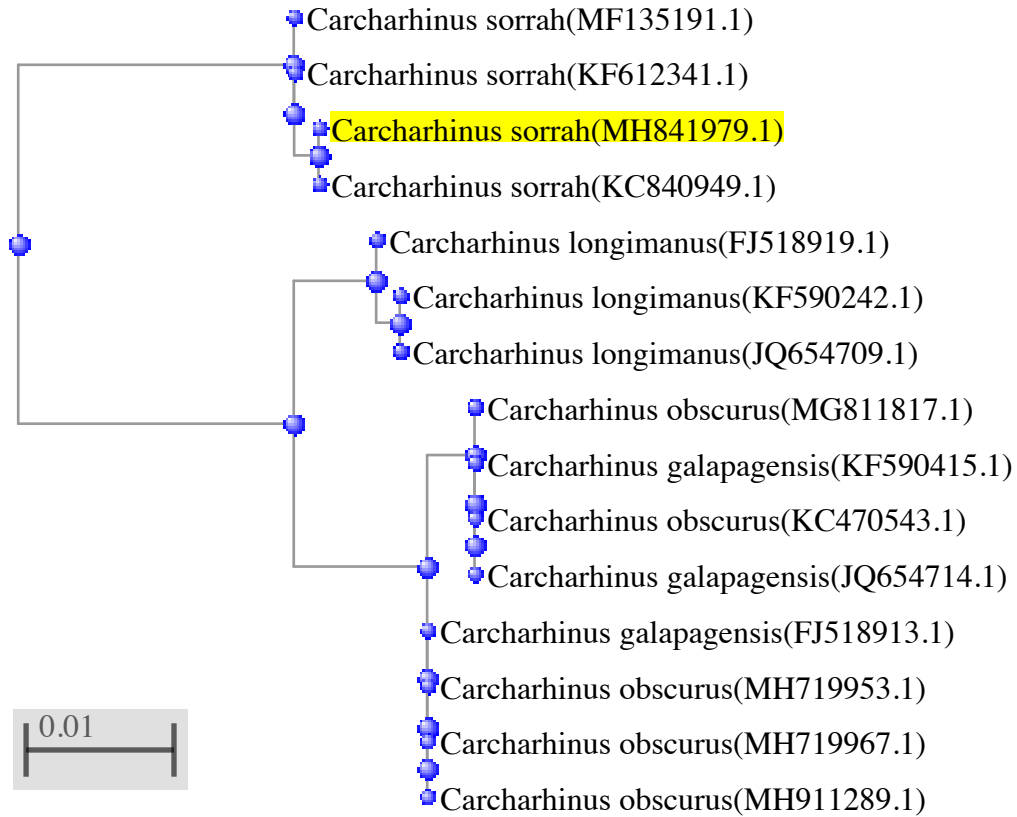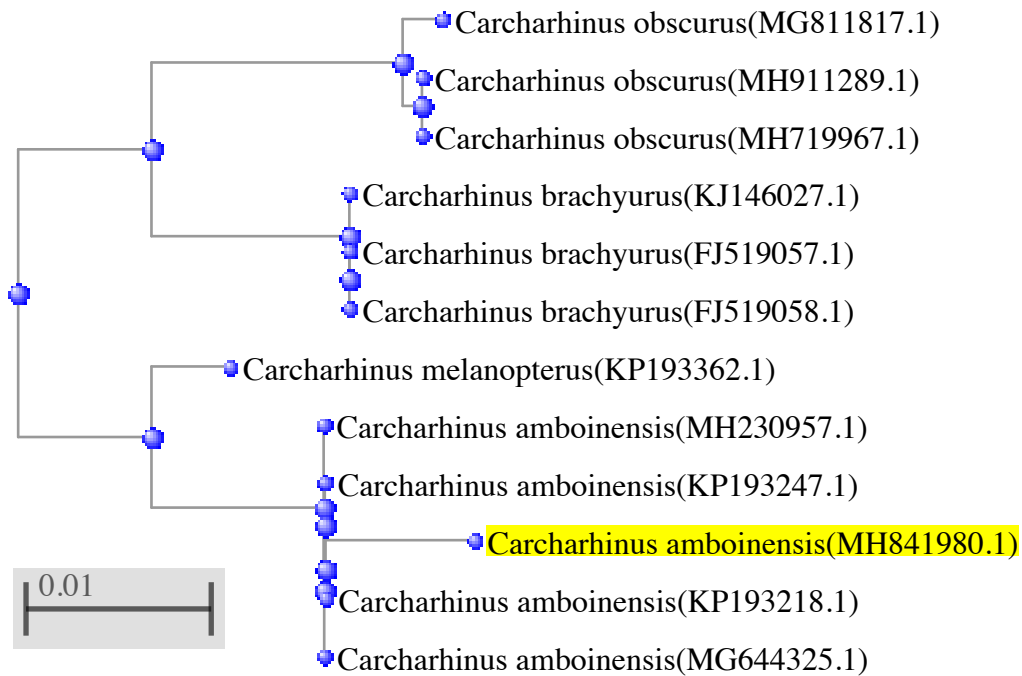

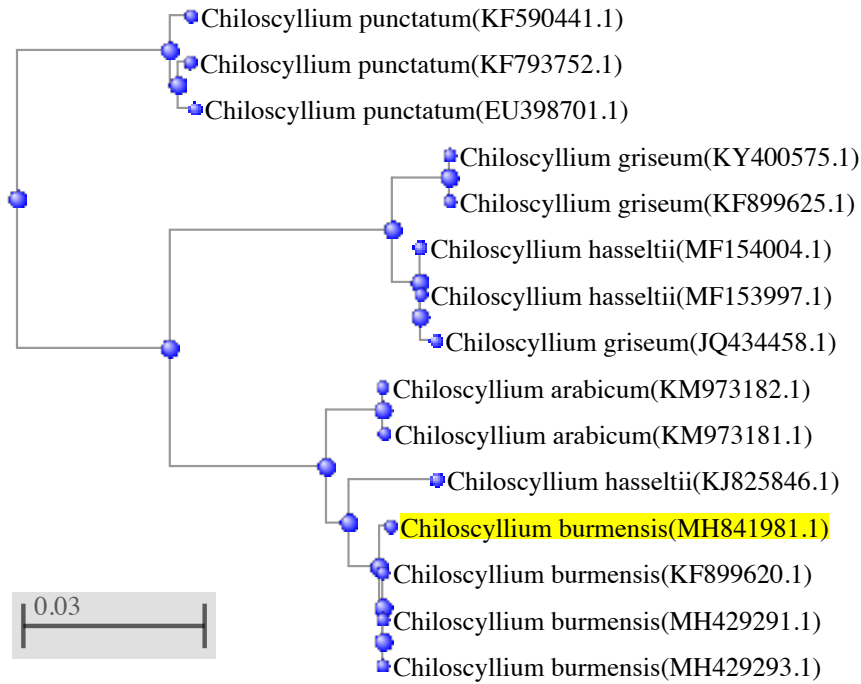

5.

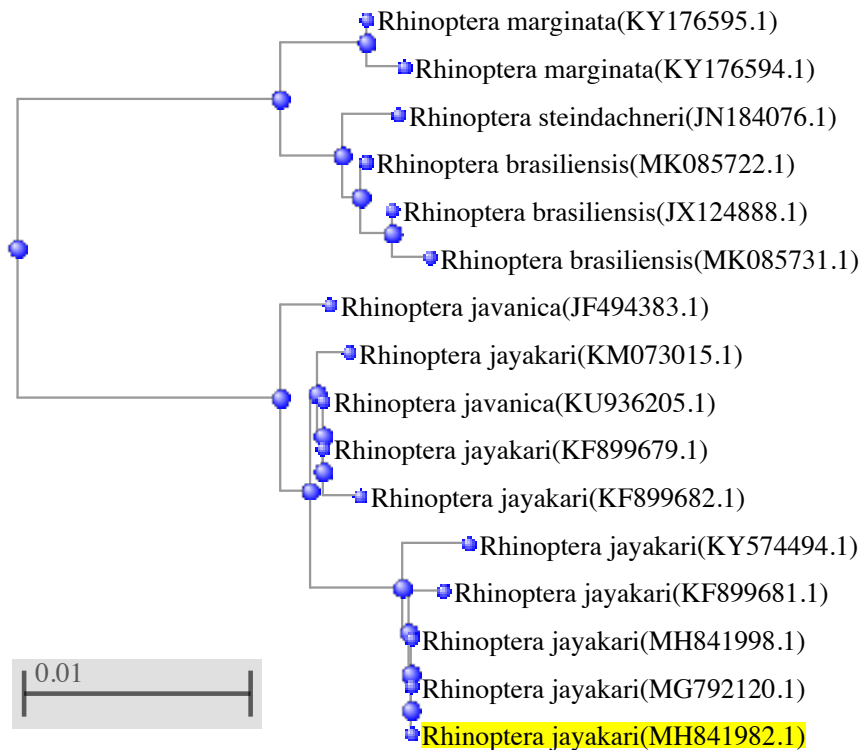

6.

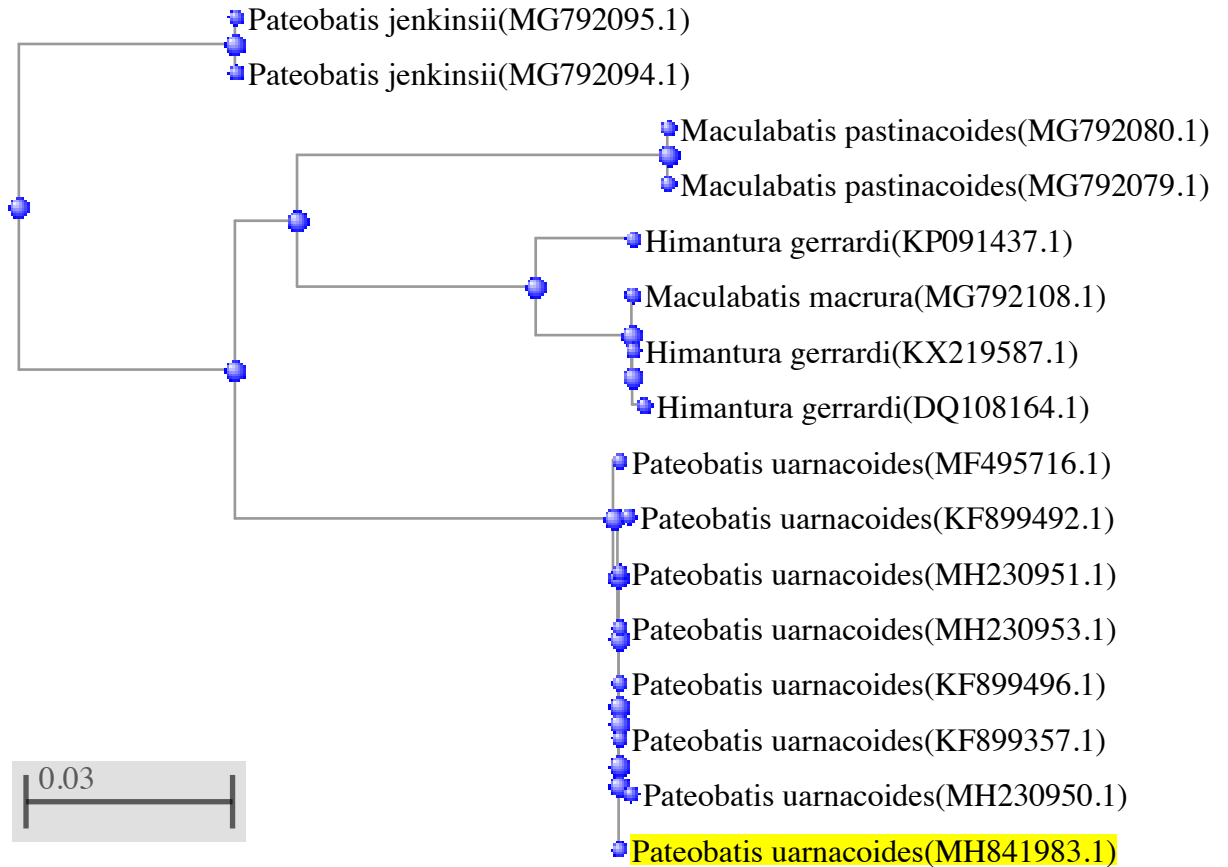

7.

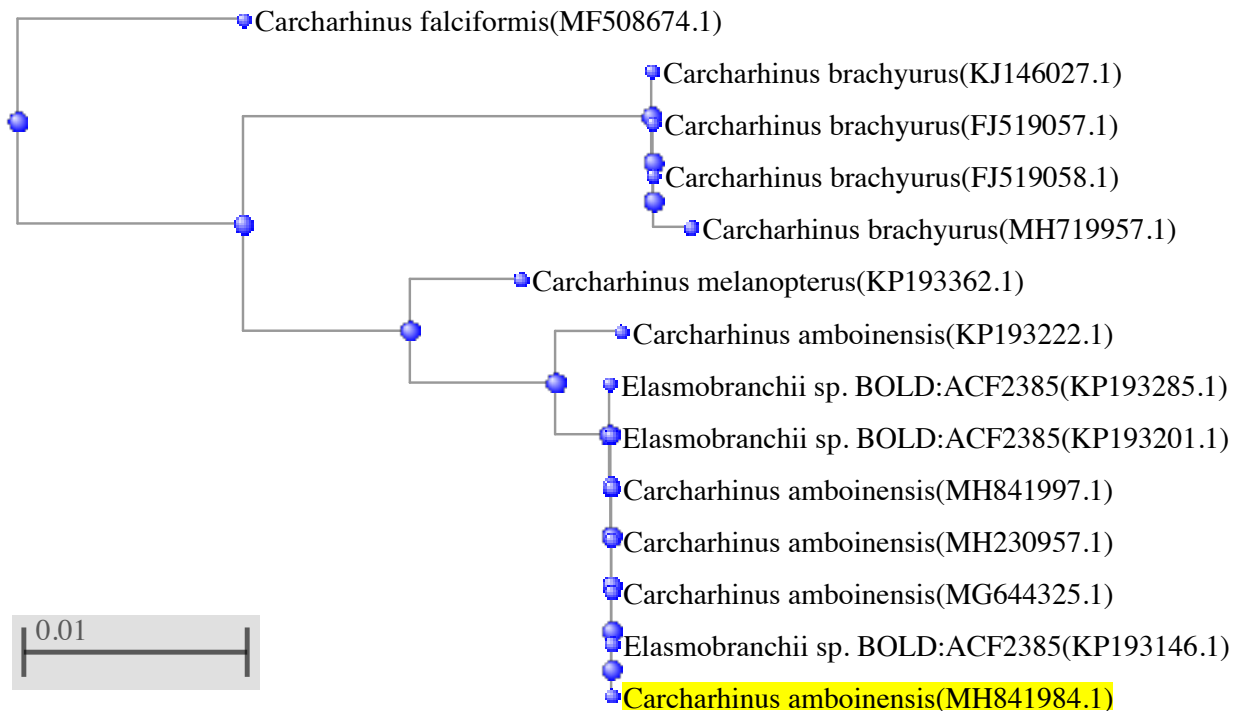

8.

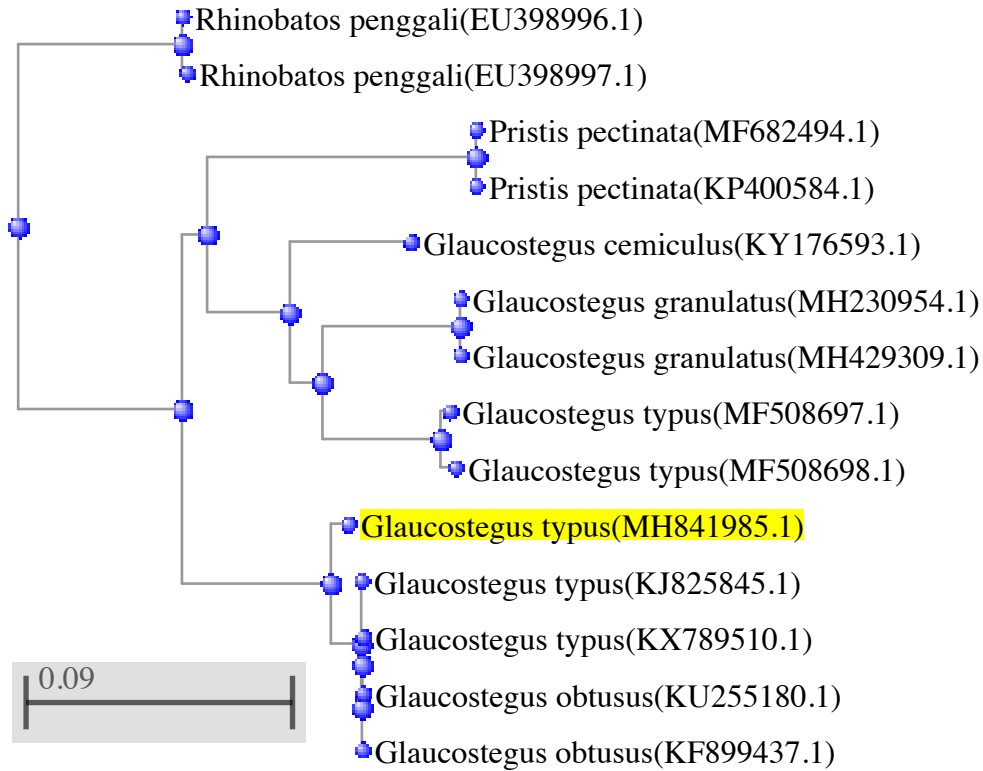

9.

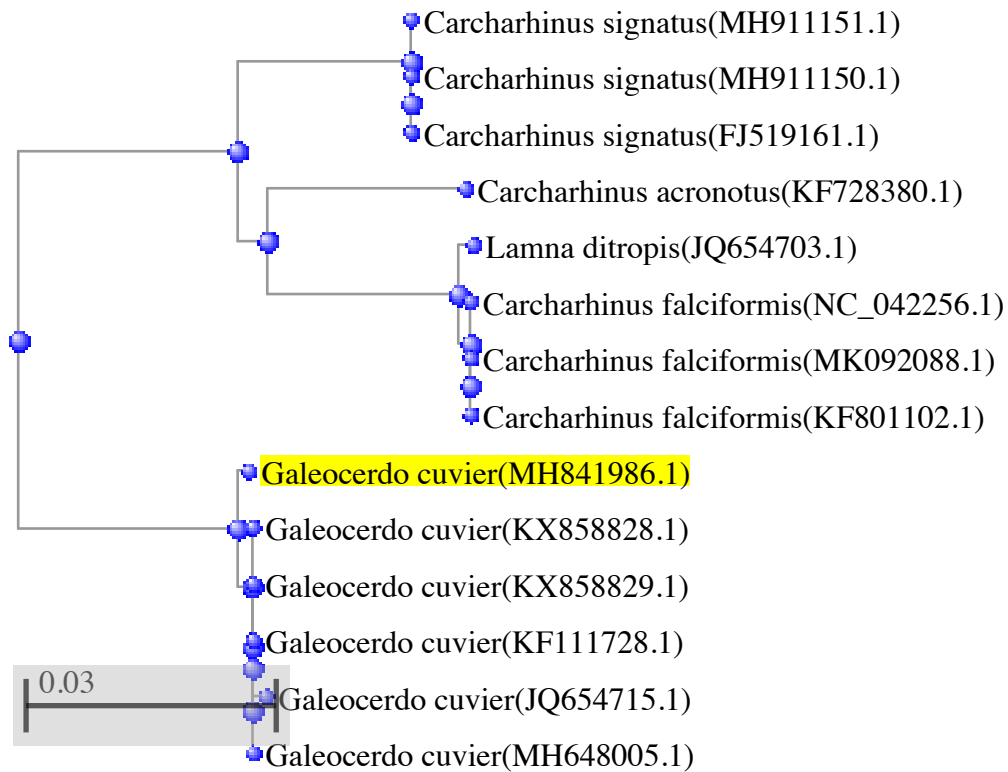

10.

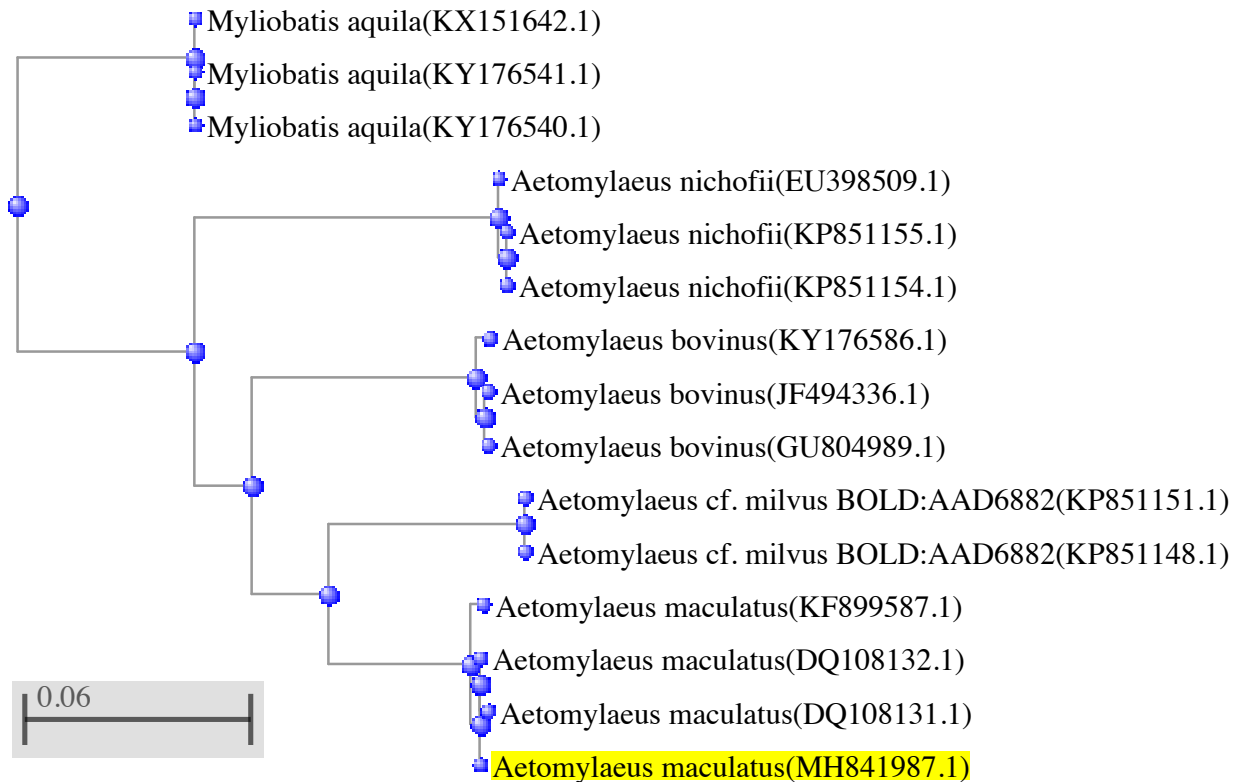

11.

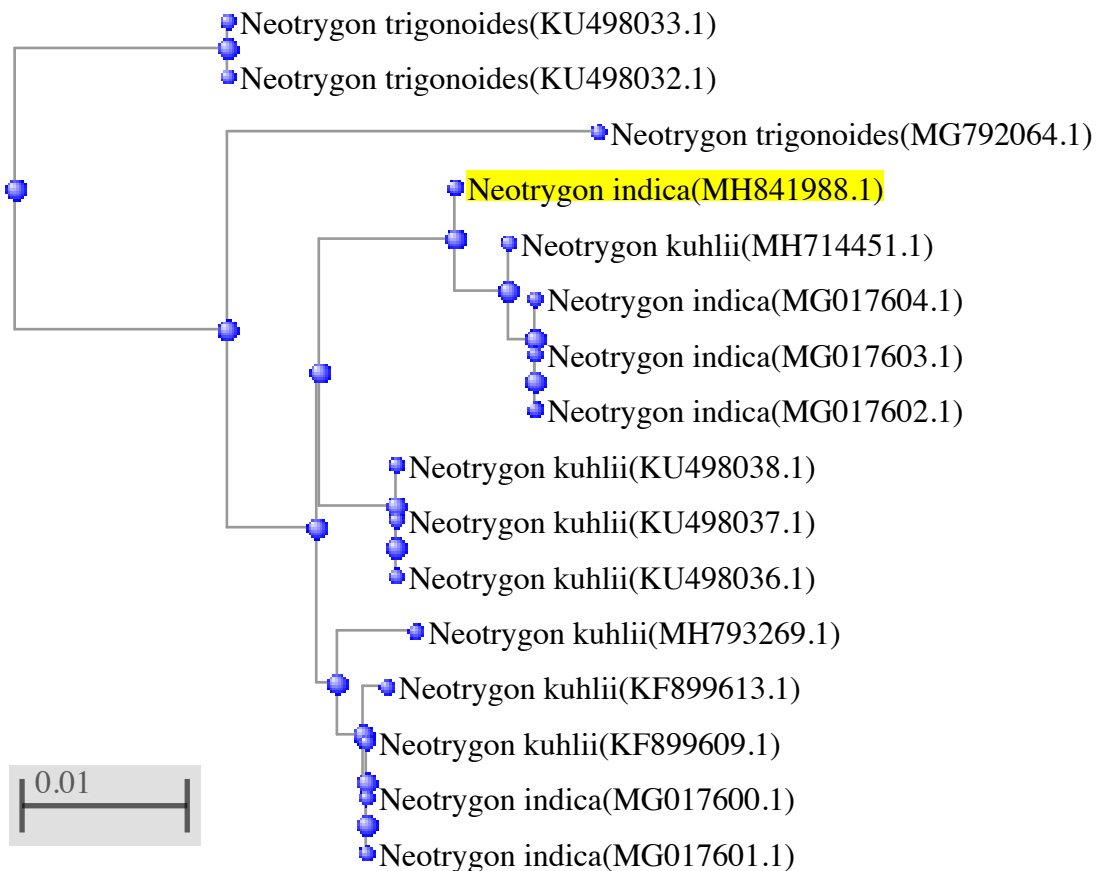

12.

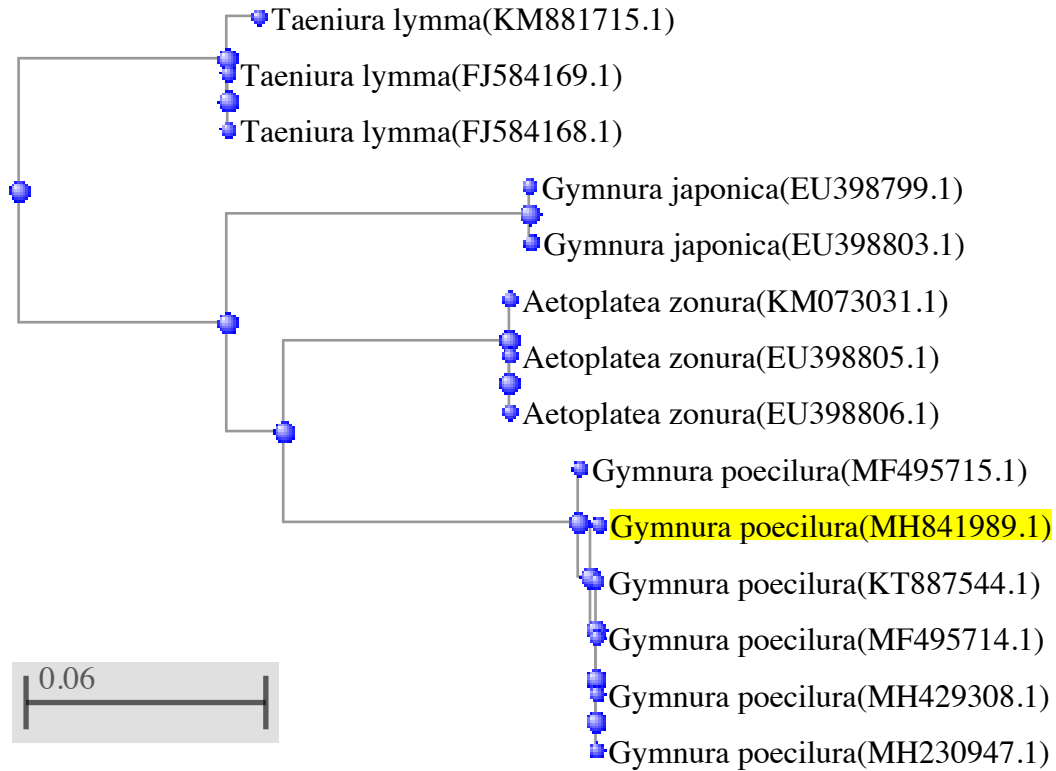

13.

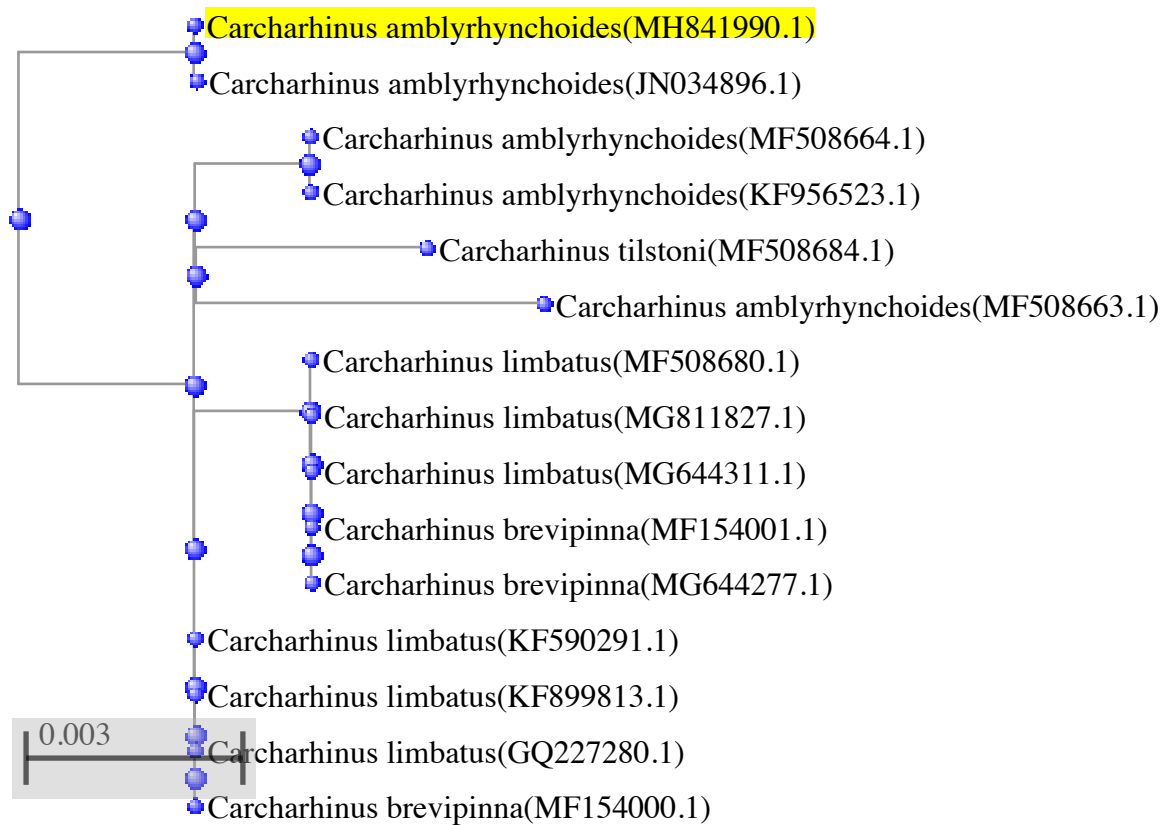

14.

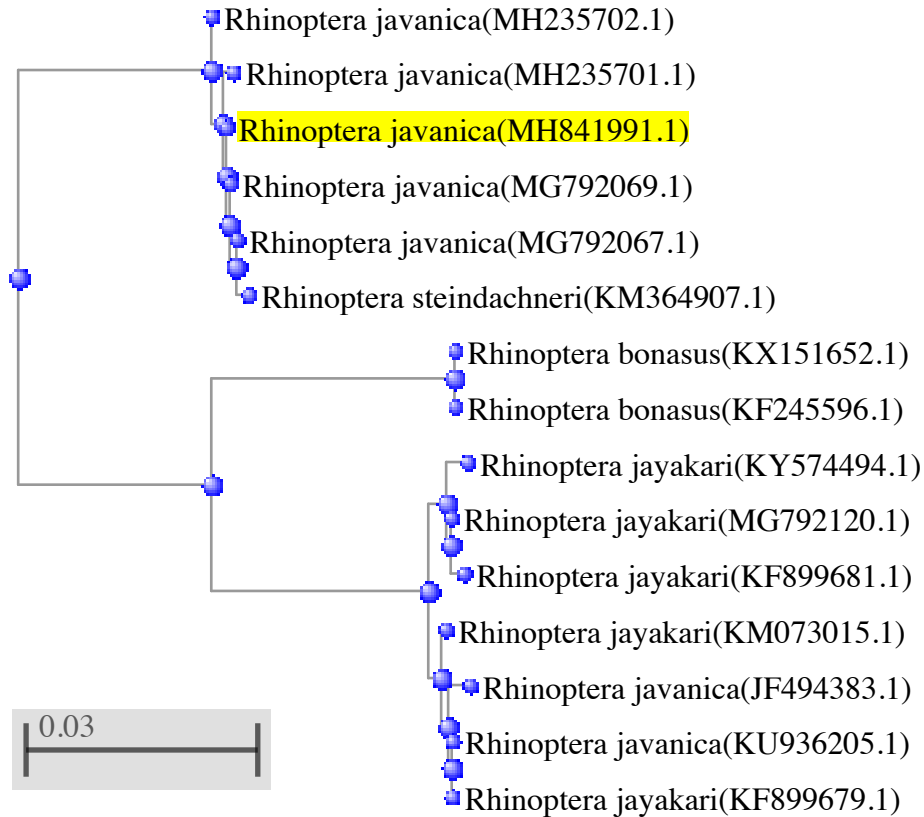

15.

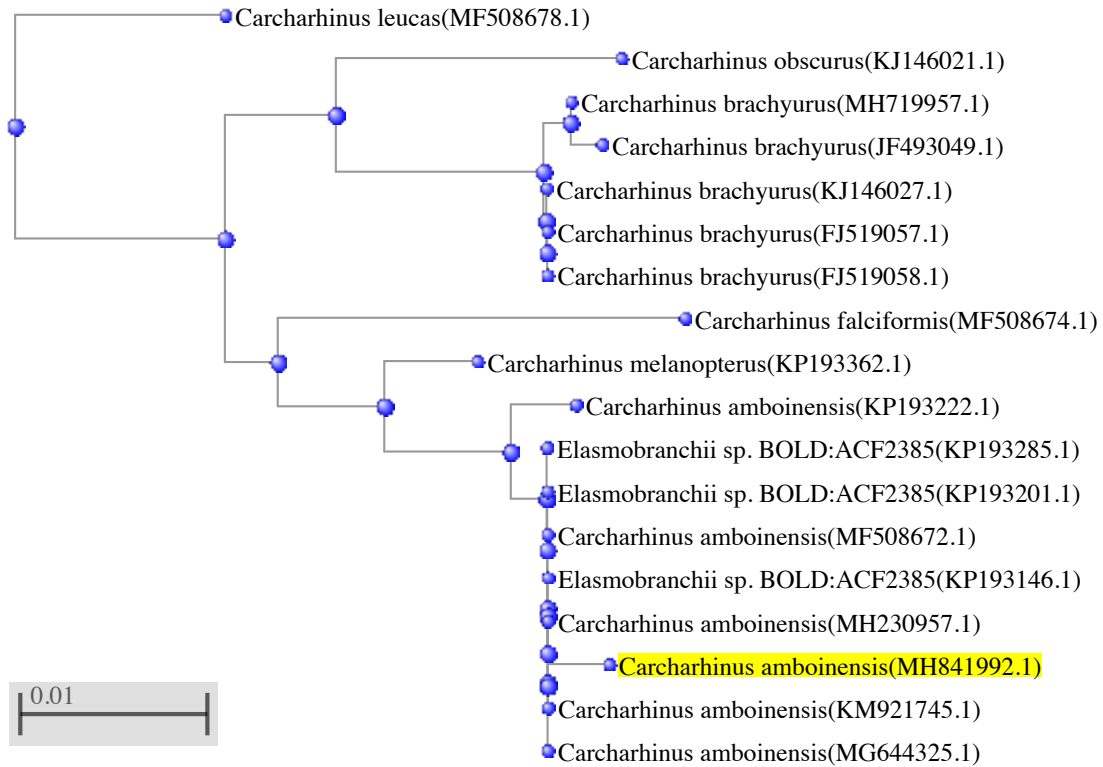

16.

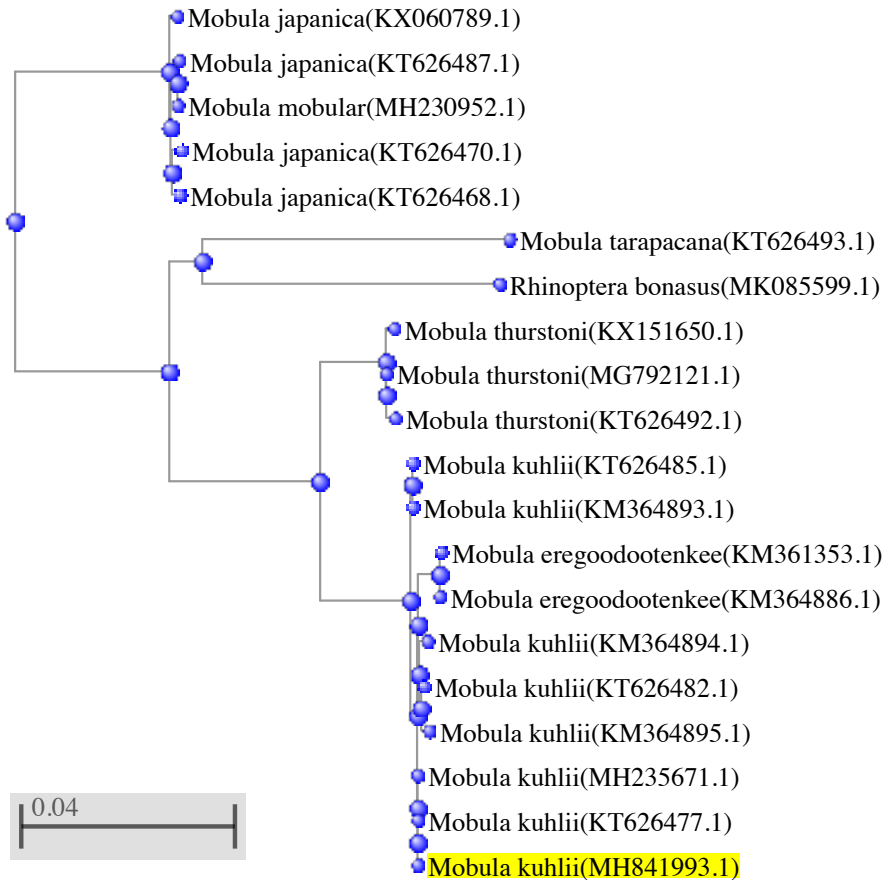

17.

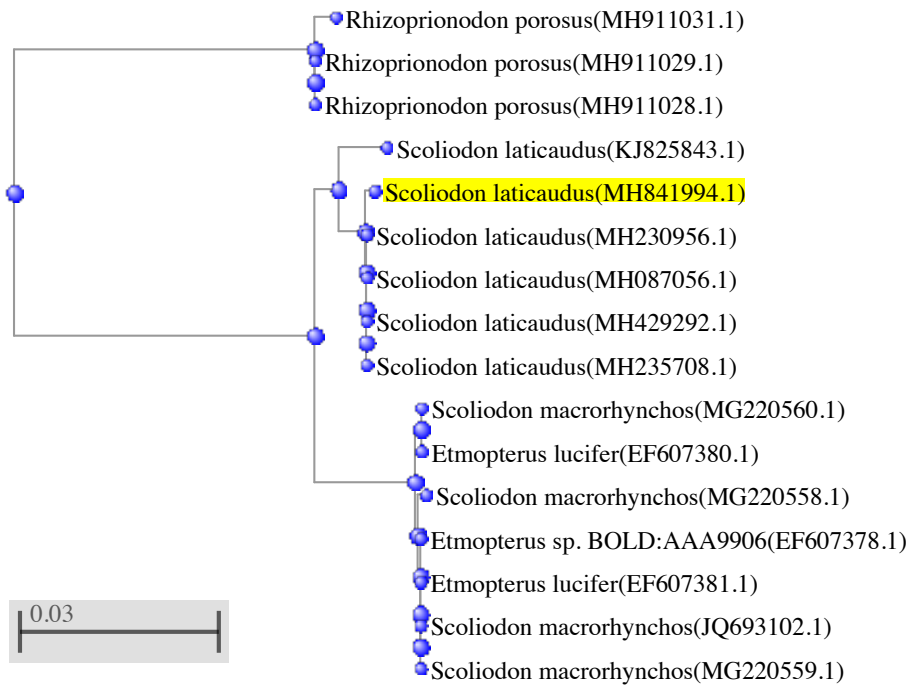

18.

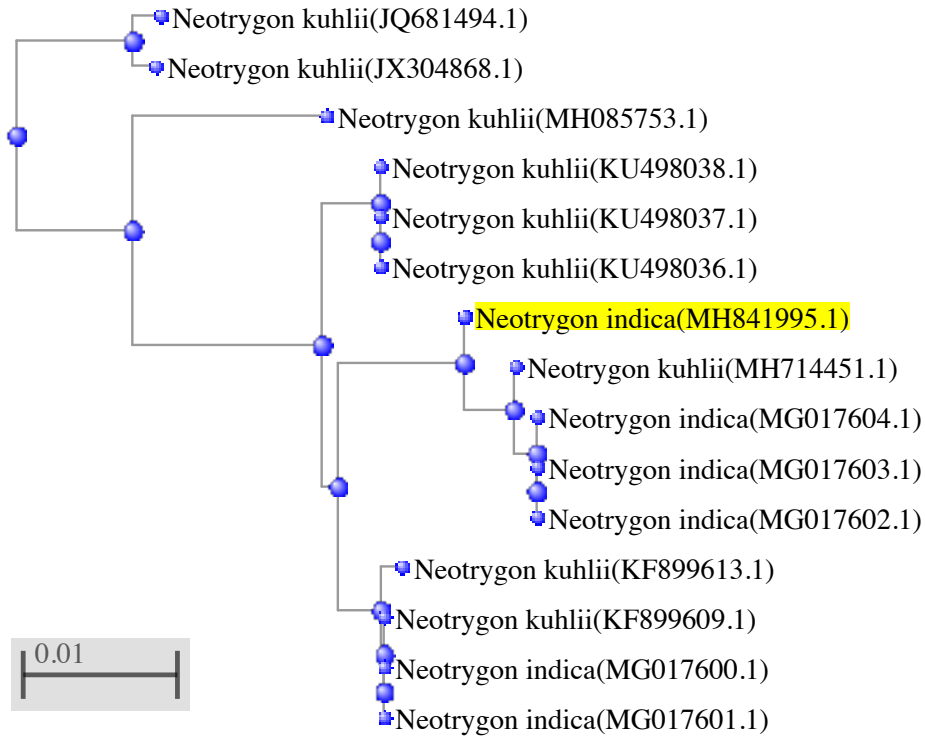

19.

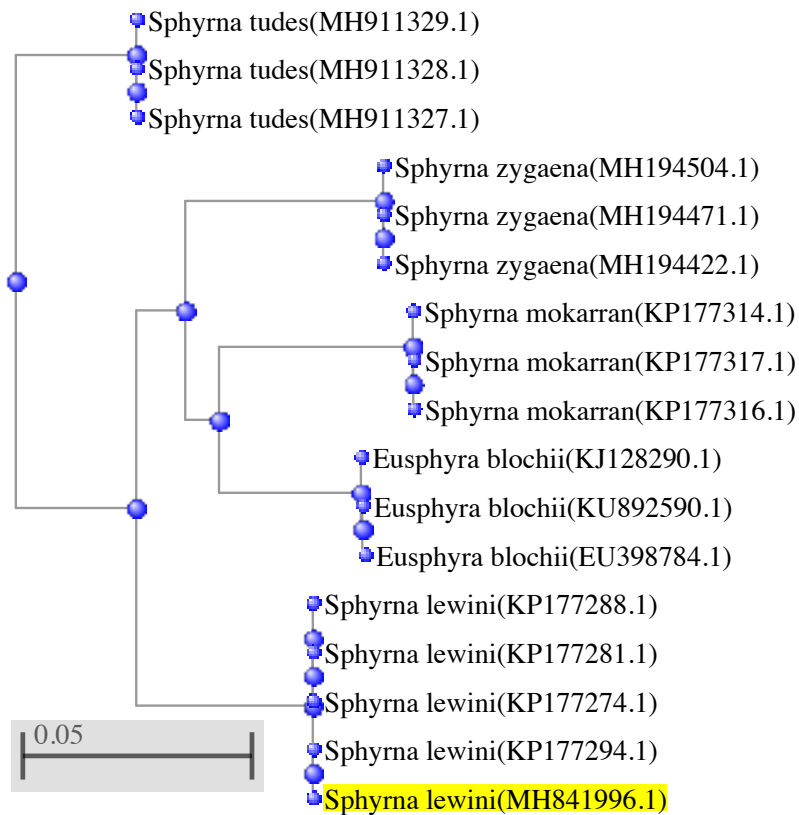

20.

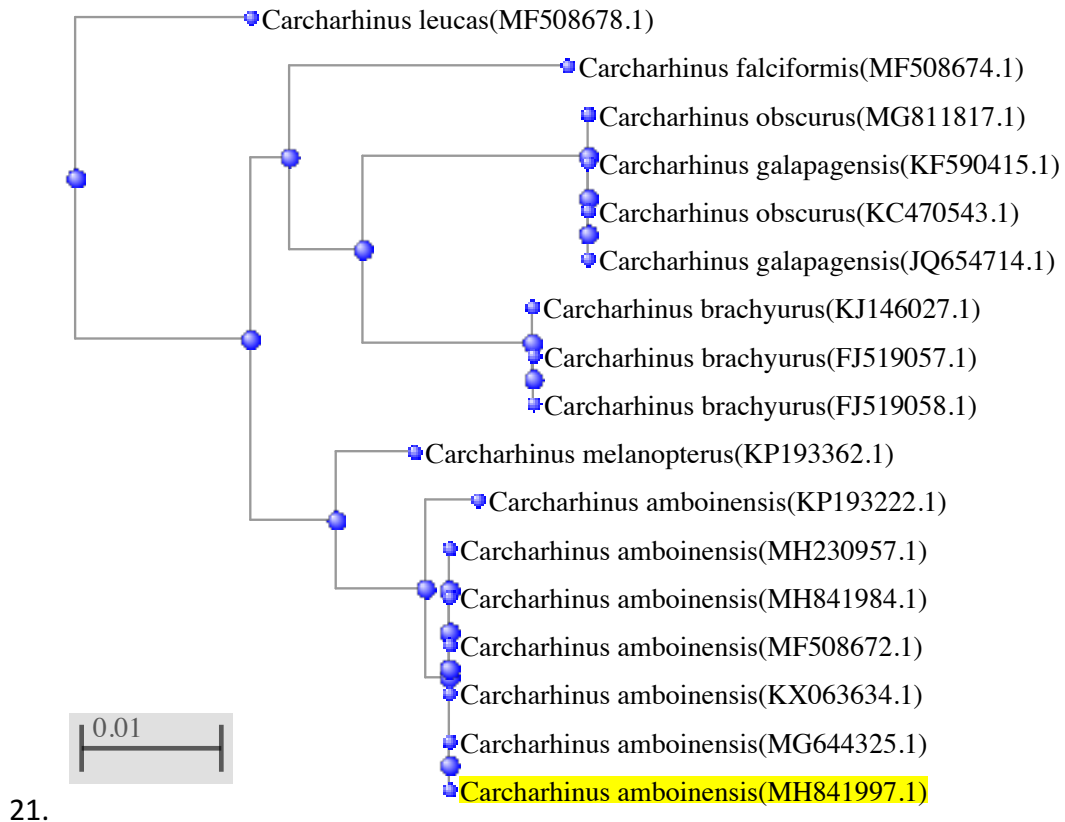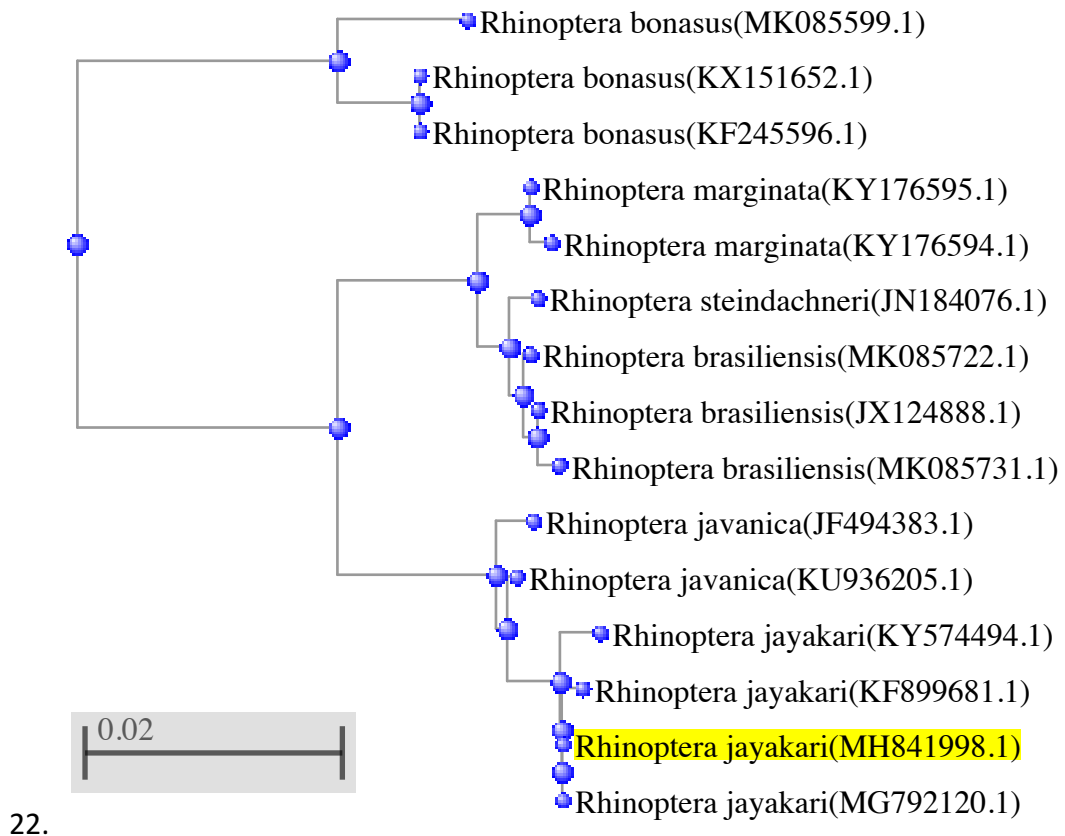

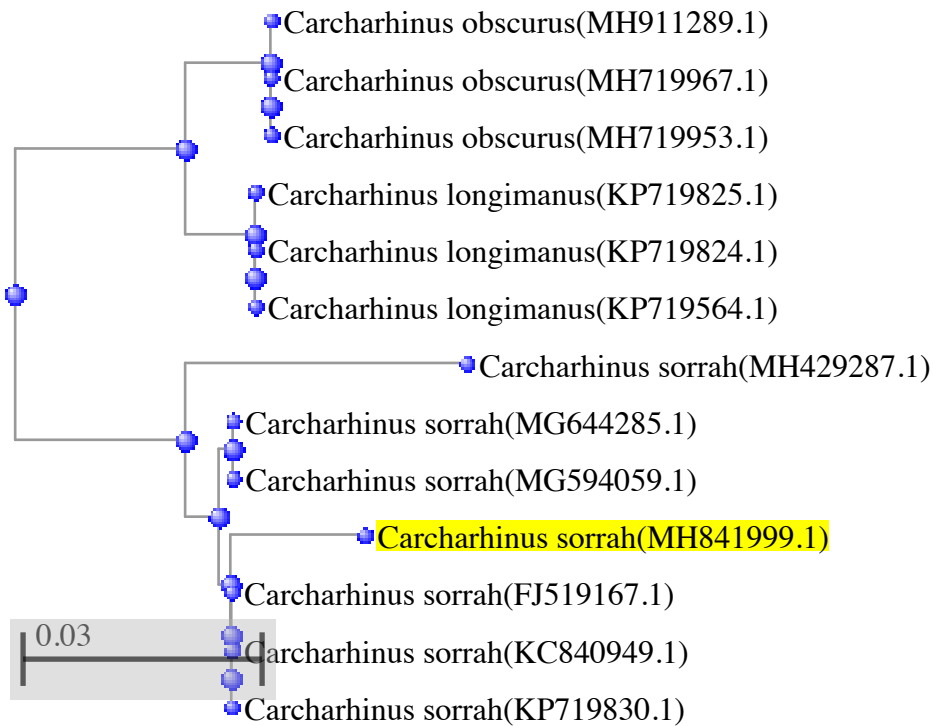

23.

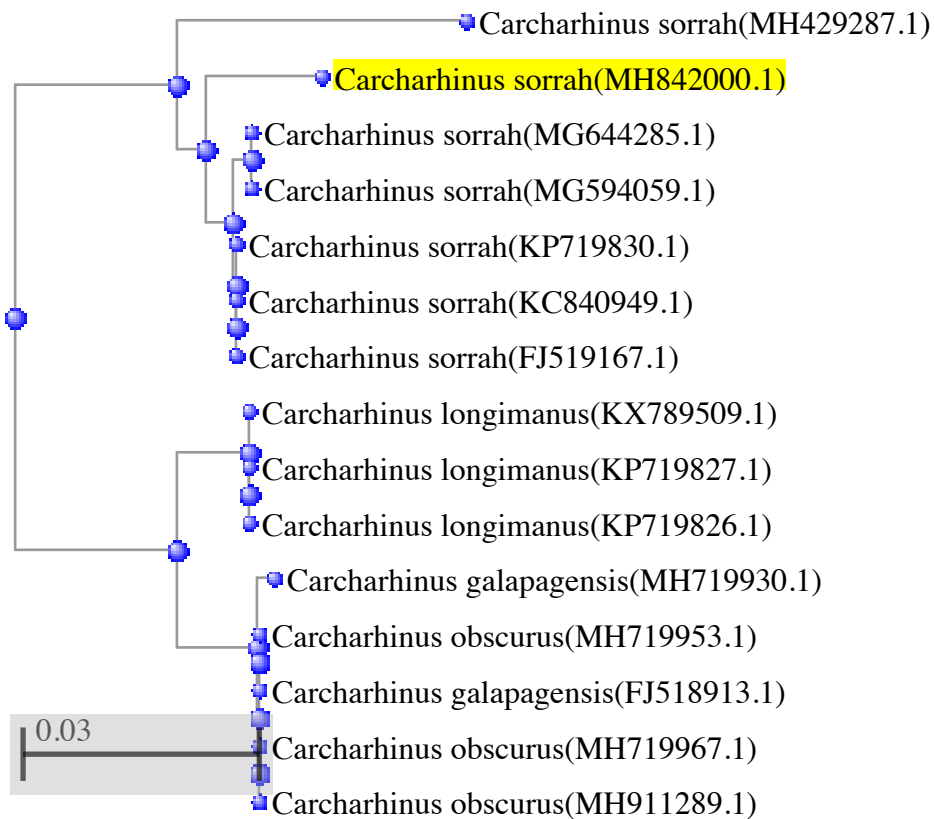

24.

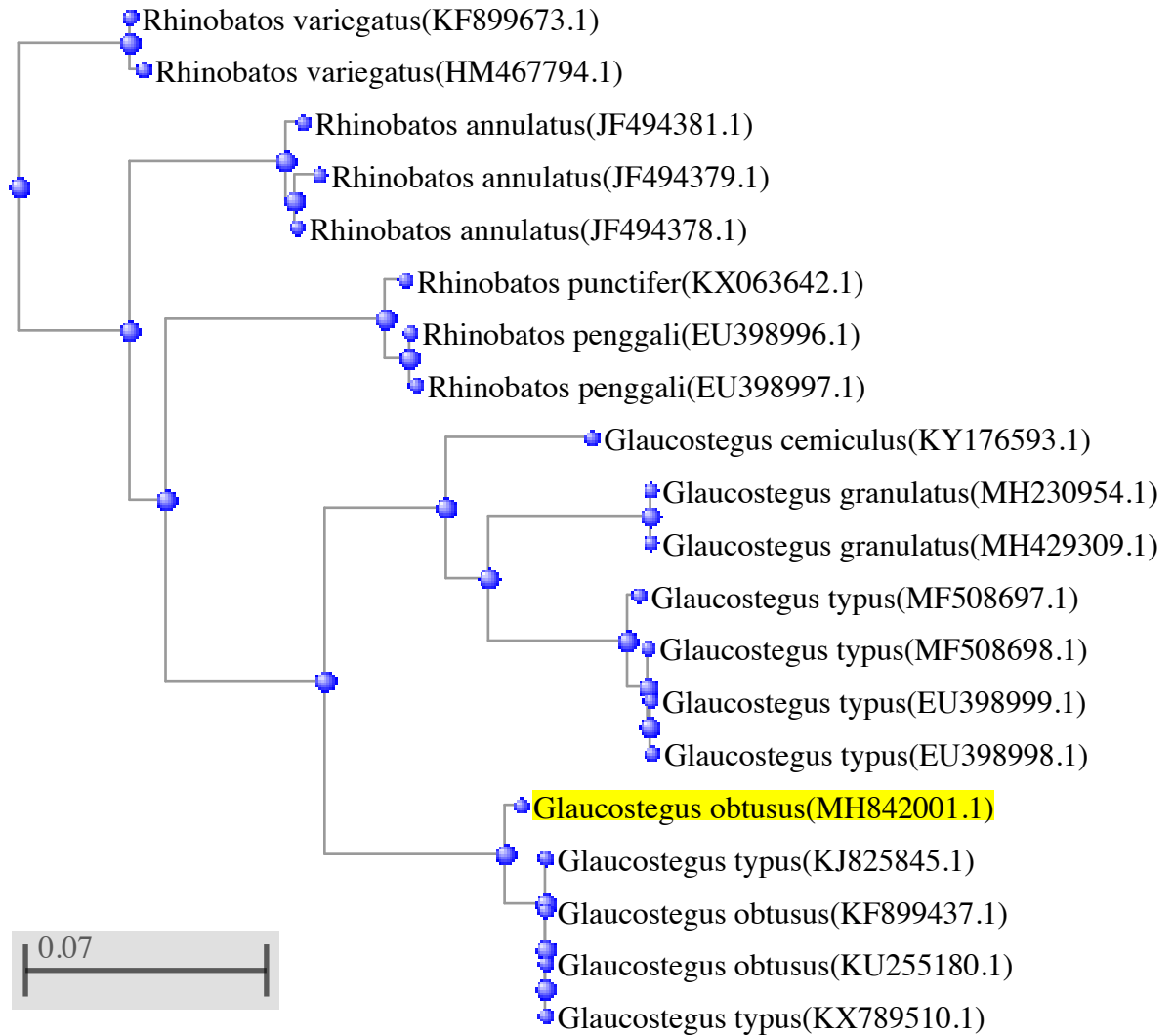

25.

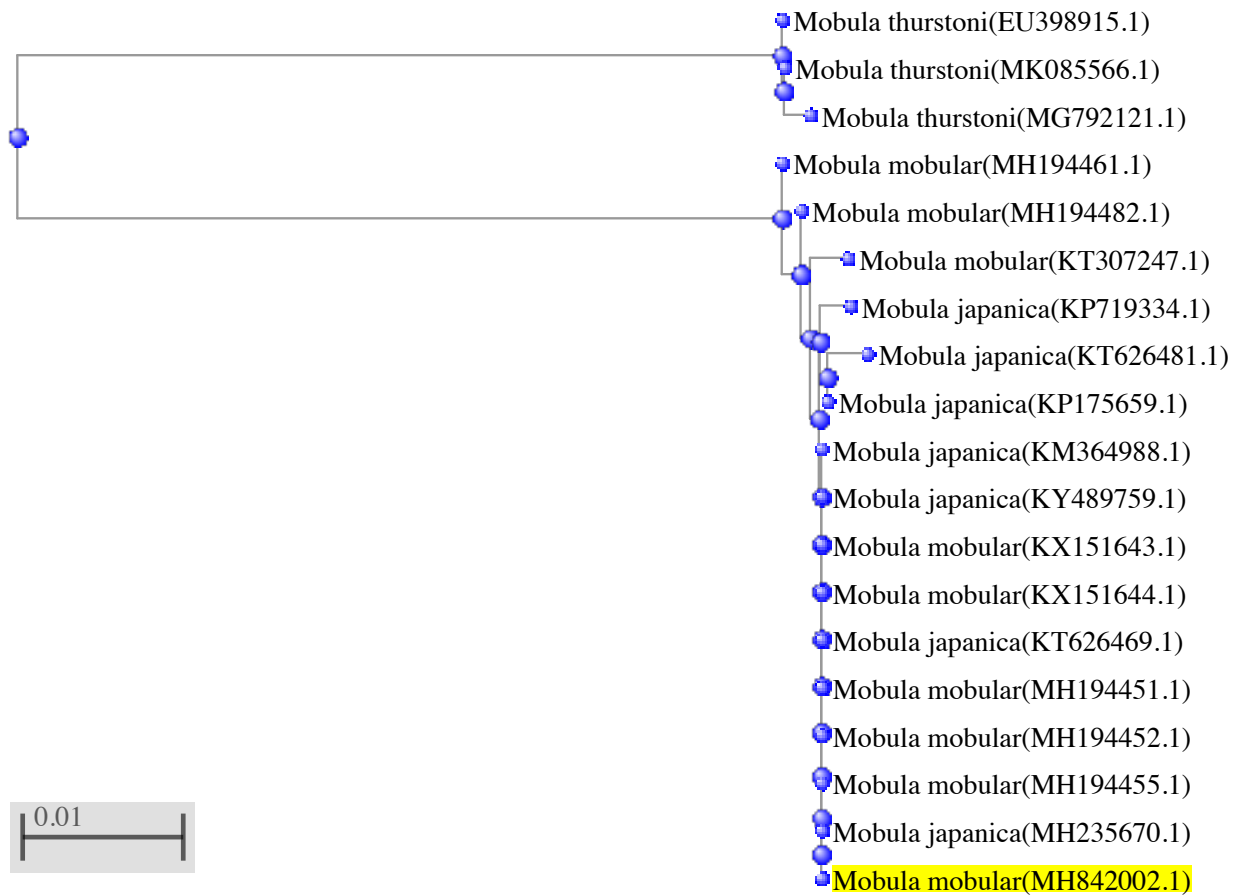

26.

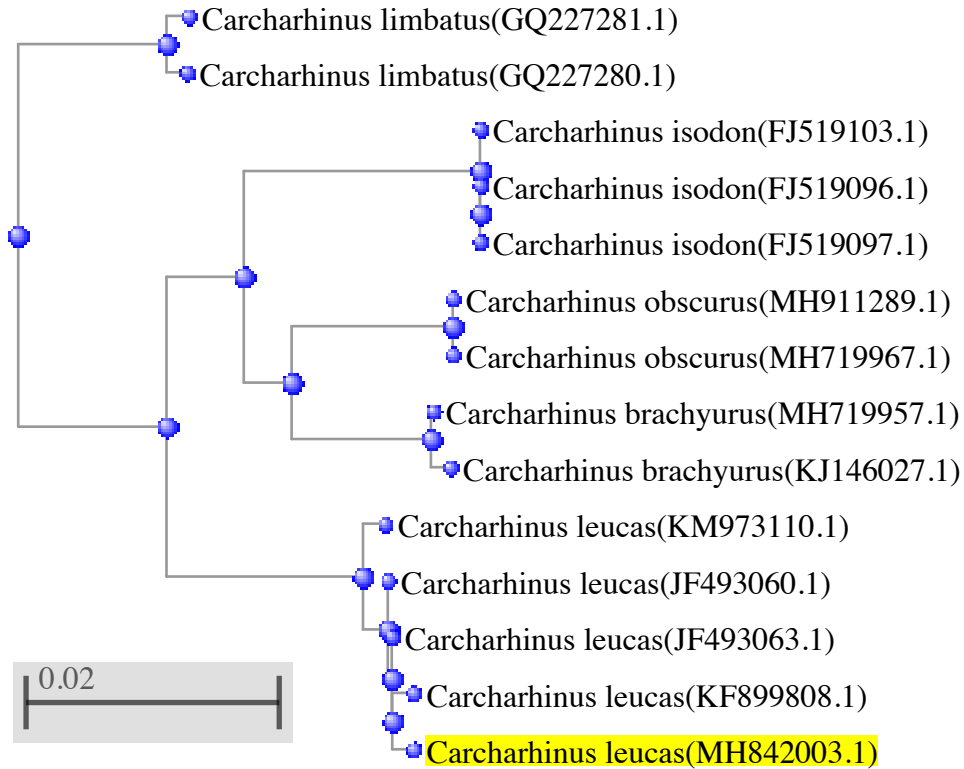

27.

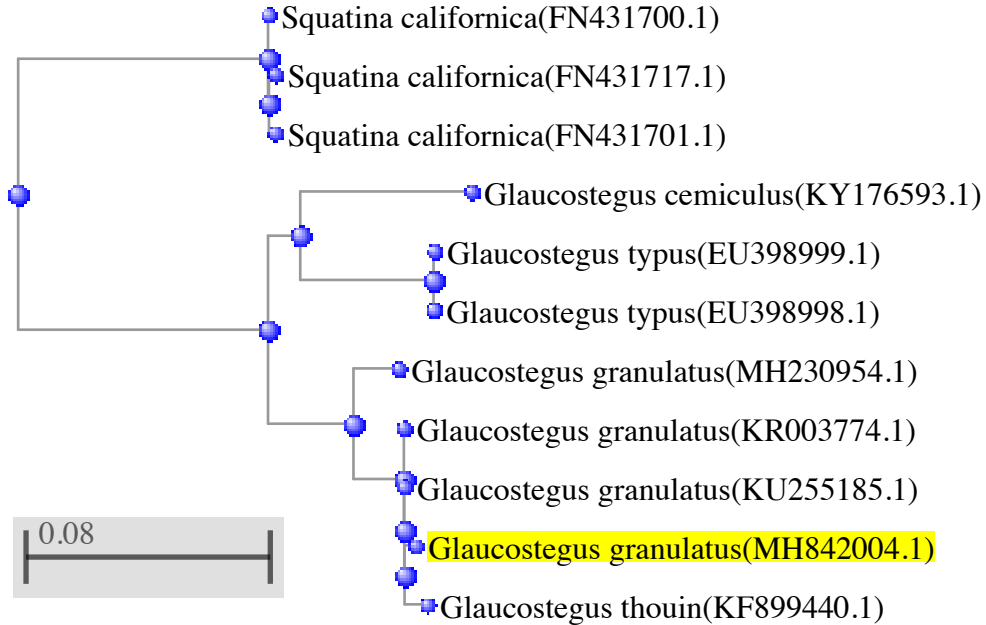

28.

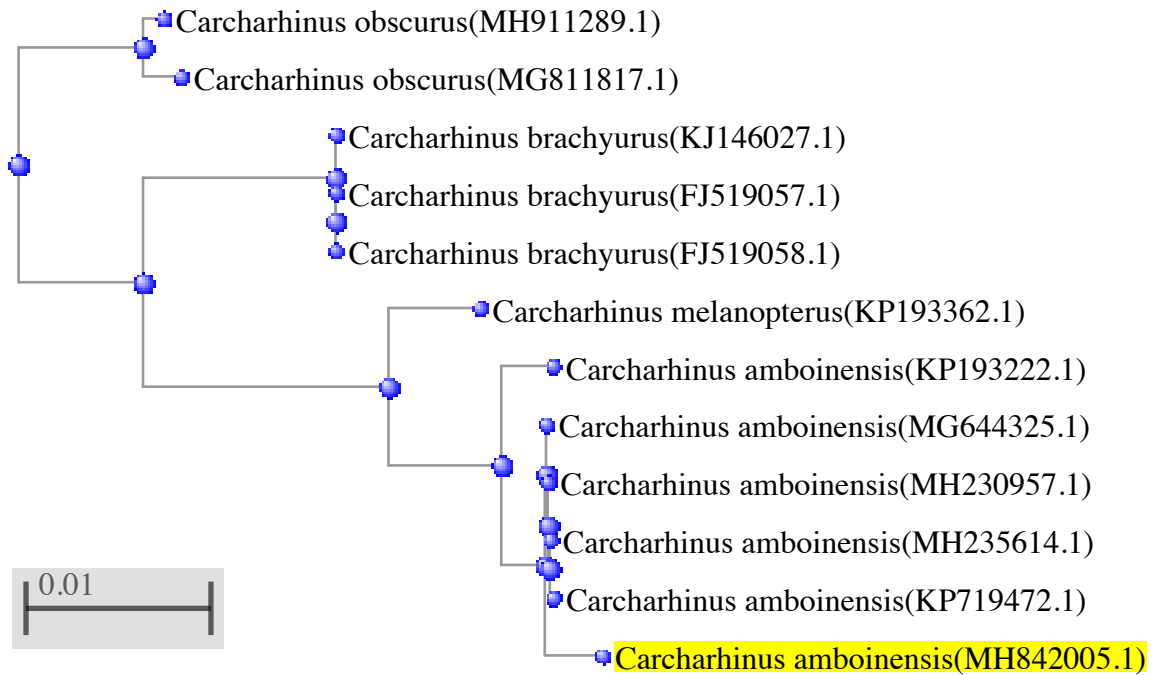

29.

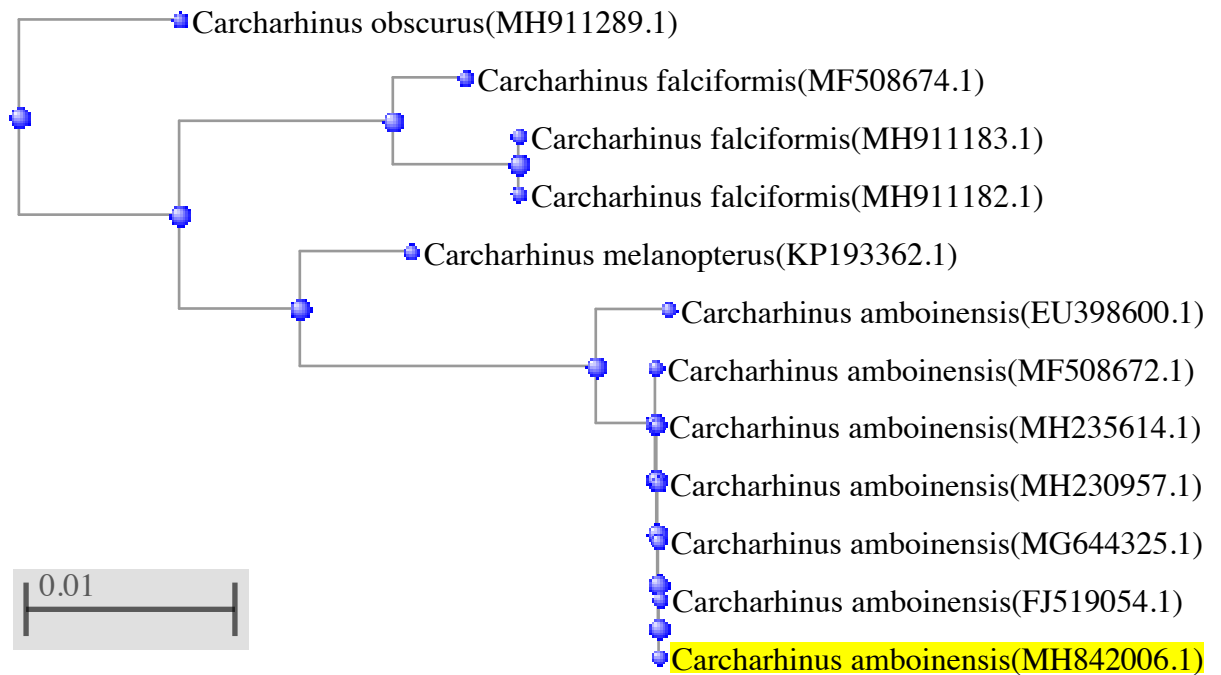

30.

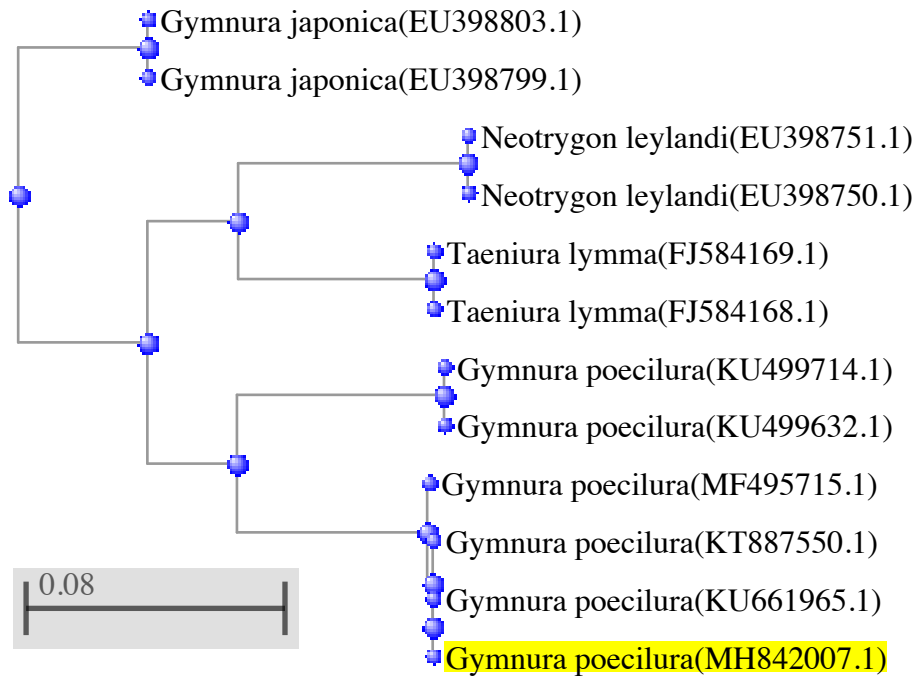

31.

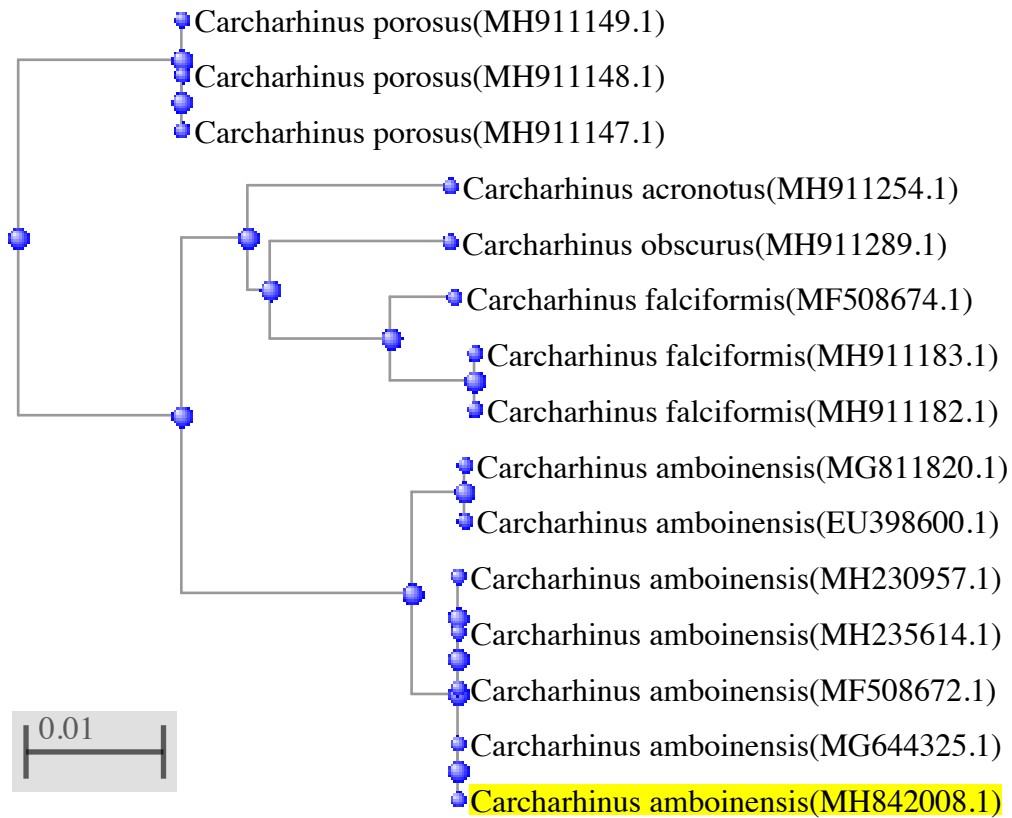

32.

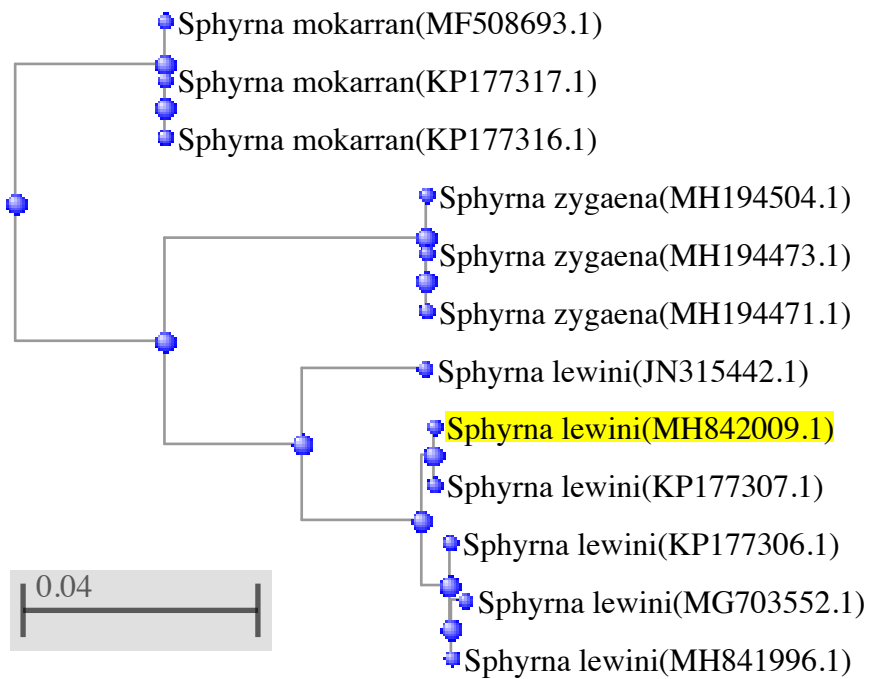

33.

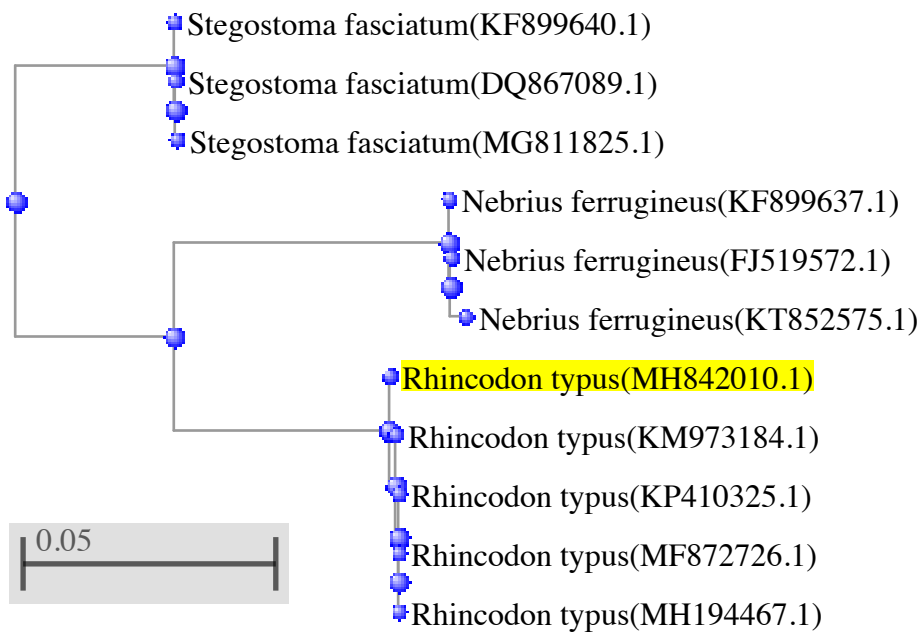

34.

Supplement: S2 Fig — Glaucostegus typus (accession number: MH841985) and Mobula mobular (accession number: MH842002) shows discrepancies with NCBI BLAST based molecular species identification (See supporting information S2 Fig). (PDF) [file pone.0222273.s002.pdf]
