# Supplementary material for: DNA analysis of elasmobranch products originating from Bangladesh reveals unregulated elasmobranch fishery and trade on species of global conservation concern
Source: PLoS One. 2019 Sep 25;14(9):e0222273. doi: 10.1371/journal.pone.0222273 (PMC6760772; doi:10.1371/journal.pone.0222273)
Supplement: S2 Table — (DOCX) [file pone.0222273.s004.docx]

Supplementary material

**Table S1. Comparative results on identification of specimens using two different databases with their accession numbers.**

| **Sl. No** | **Accession**  **number** | **BP** | **Identification using NCBI’s BLAST tool** | **Identification using BoLD database** |
| --- | --- | --- | --- | --- |
| 1 | MH817051 | 650 | *Chiloscyllium burmensis* | *Chiloscyllium burmensis* |
| 3 | \| MH841978 \|  \| \| --- \| --- \| | 621 | *Carcharhinus brevipinna* | *Carcharhinus brevipinna* |
| 4 | MH841979 | 627 | *Carcharhinus sorrah* | *Carcharhinus sorrah* |
| 5 | MH841980 | 630 | *Carcharhinus amboinensis* | *Carcharhinus amboinensis* |
| 6 | MH841981 | 432 | *Chiloscyllium burmensis* | *Chiloscyllium burmensis* |
| 7 | MH841982 | 618 | *Rhinoptera jayakari* | *Rhinoptera jayakari* |
| 8 | MH841983 | 622 | *Pateobatis uarnacoides* | *Pateobatis uarnacoides* |
| 9 | MH841984 | 615 | *Carcharhinus amboinensis* | *Carcharhinus amboinensis* |
| 10 | MH841985 | 624 | *This specimen is more closely resembles with* ***G. obtusus*** *using the BoLD database* | *Glaucostegus typus* |
| 11 | MH841986 | 649 | *Galeocerdo cuvier* | *Galeocerdo cuvier* |
| 12 | MH841987 | 521 | *Aetomylaeus maculatus* | *Aetomylaeus maculatus* |
| 13 | MH841988 | 630 | *Neotrygon indica* | *Neotrygon indica* |
| 14 | MH841989 | 613 | *Gymnura poecilura* | *Gymnura poecilura* |
| 15 | MH841990 | 629 | *Carcharhinus amblyrhynchoides* | *Carcharhinus amblyrhynchoides* |
| 16 | MH841991 | 620 | *Rhinoptera javanica* | *Rhinoptera javanica* |
| 17 | MH841992 | 621 | *Carcharhinus amboinensis* | *Carcharhinus amboinensis* |
| 18 | MH841993 | 636 | *Mobula kuhlii* | *Mobula kuhlii* |
| 19 | MH841994 | 621 | *Scoliodon laticaudus* | *Scoliodon laticaudus* |
| 20 | MH841995 | 643 | *Neotrygon indica* | *Neotrygon indica* |
| 21 | MH841996 | 634 | *Sphyrna lewini* | *Sphyrna lewini* |
| 22 | MH841997 | 615 | *Carcharhinus amboinensis* | *Carcharhinus amboinensis* |
| 23 | MH841998 | 618 | *Rhinoptera jayakari* | *Rhinoptera jayakari* |
| 24 | MH841999 | 417 | *Carcharhinus sorrah* | *Carcharhinus sorrah* |
| 25 | MH842000 | 417 | *Carcharhinus sorrah* | *Carcharhinus sorrah* |
| 26 | MH842001 | 555 | *Glaucostegus obtusus* | *Glaucostegus obtusus* |
| 27 | MH842002 | 635 | *This specimen is more closely resembles with* ***M. japanica*** *using the BoLD database* | *Mobula mobular* |
| 28 | MH842003 | 600 | *Carcharhinus leucas* | *Carcharhinus leucas* |
| 29 | MH842004 | 558 | *Glaucostegus granulatus* | *Glaucostegus granulatus* |
| 30 | MH842005 | 642 | *Carcharhinus amboinensis* | *Carcharhinus amboinensis* |
| 31 | MH842006 | 320 | *Carcharhinus amboinensis* | *Carcharhinus amboinensis* |
| 32 | MH842007 | 461 | *Gymnura poecilura* | *Gymnura poecilura* |
| 33 | MH842008 | 281 | *Carcharhinus amboinensis* | *Carcharhinus amboinensis* |
| 34 | MH842009 | 603 | *Sphyrna lewini* | *Sphyrna lewini* |
| 35 | MH842010 | 600 | *Rhincodon typus* | *Rhincodon typus* |
